# Supplementary material for: Autocrine interferon poisoning mediates ADAR1-dependent synthetic lethality in BRCA1/2-mutant cancers
Source: Nat Commun. 2025 Jul 29;16:6972. doi: 10.1038/s41467-025-62309-5 (PMC12307730; doi:10.1038/s41467-025-62309-5)
Supplement: Supplementary file 1 — Supplementary Information [file 41467_2025_62309_MOESM1_ESM.pdf]

Supplementary Information

Autocrine interferon poisoning mediates ADAR1-dependent synthetic lethality in *BRCA1/2*-mutant cancers

Roman M Chabanon[1,2] *, Liudmila Shcherbakova[1], Magali Lacroix-Triki[3,4], Marine Aglave[5], Jean Zeghondy[6], Victor Kriaa[7], Antoine Gougé[1], Marlène Garrido[1], Elodie Edmond[8], Ludovic Bigot[9], Dragomir B Krastev[2], Rachel Brough[2], Stephen J Pettitt[2], Thibault Thomas-Bonafos[10], Robert Samstein[11,12,13], Christophe Massard[10], Marc Deloger[5], Andrew NJ Tutt[14,15], Fabrice Barlesi[6], Yohann Loriot[9,10], Suzette Delaloge[6], Marcel Tawk[7], Cindy Degerny[7], Yea-Lih Lin[16], Barbara Pistilli[6], Philippe Pasero[16], Christopher J Lord[2] * and Sophie Postel-Vinay[1,10,17] *

[1]The ATIP-Avenir Inserm and ERC StG (Epi)genetic Vulnerabilities in Solid Tumors and Sarcoma Laboratory, Inserm Unit UMR981, Université Paris-Saclay, Gustave Roussy, Villejuif, France; [2]The CRUK Gene Function Laboratory and Breast Cancer Now Toby Robins Breast Cancer Research Centre, The Institute of Cancer Research, London, UK; [3]Molecular Characterization of Breast and Gynecological Cancers Team, Inserm Unit UMR981, Université Paris-Saclay, Gustave Roussy, Villejuif, France; [4]Department of Pathology, Gustave Roussy, Villejuif, France; [5]Bioinformatics (BiGR) Platform, Gustave Roussy, Villejuif, France; [6]Department of Medical Oncology, Gustave Roussy, Villejuif, France; [7]Inserm Unit U1195, University Paris-Saclay, Le Kremlin Bicêtre, France. [8]Experimental and Translational Pathology (PETRA) Platform, AMMICa Unit (CNRS Unit UMS3655, Inserm Unit US23), Université Paris-Saclay, Gustave Roussy, Villejuif, France; [9]Adaptive Resistance to Anti-Cancer Therapies Team, Inserm Unit UMR981, Université Paris-Saclay, Gustave Roussy, Villejuif, France; [10]Drug Development Department (DITEP), Gustave Roussy, Villejuif, France; [11]Department of Radiation Oncology, Memorial Sloan Kettering Cancer Center, New York, USA; [12]Department of Radiation Oncology, Mount Sinai Hospital, New York, USA; [13]Precision Immunology Institute at Icahn School of Medicine at Mount Sinai, New York, USA; [14]The Breast Cancer Now Toby Robins Breast Cancer Research Centre, The Institute of Cancer Research, London, UK; [15]The Breast Cancer Now Research Unit, Guy's Hospital Cancer Centre, King's College London, UK; [16]Maintenance of Genome Integrity during DNA Replication Team, CNRS Unit UMR9002, Université de Montpellier, Institut de Génétique Humaine, Montpellier, France; [17]University College of London, Cancer Institute, London, UK.

This PDF file contains:

- **Supplementary Tables.** Supplementary Tables 1-4, related to the Methods (pages 2-6).
- **Supplementary Figures.** Supplementary Figures 1-10 and associated legends, related to the main Figures (pages 7-35).

Supplementary Tables

Supplementary Table 1. List of antibodies used in this study.

Target	Epitope specificity	Application	Manufacturer	Reference	Working dilution
ADAR1	p110, p150	WB	Cell Signaling Technologies	#14175	1:1,000
	p110, p150	IHC	Santa Cruz	#sc-73408	1:700
	p150	IHC	Bethyl Laboratories	#A303-883A	1 :1,000
β-Actin		WB	Sigma Aldrich	#A1978	1:10,000
BRCA1		WB	Cell Signaling Technologies	#9010	1:1,000
BRCA2		WB	Cell Signaling Technologies	#10741	1:1,000
Casp3		WB	Cell Signaling Technologies	#9668	1:1,000
c-Casp3	N175	WB	Cell Signaling Technologies	#9661	1:1,000
CCNA2		IF	Abcam	#ab181591	1:1,000
CGAS		WB	Cell Signaling Technologies	#15102	1:1,000
CHK1		WB	Cell Signaling Technologies	#2360	1:1,000
p-CHK1	S345	WB	Cell Signaling Technologies	#2341	1:1,000
eIF2α		WB	Cell Signaling Technologies	#5324	1:1,000
p-eIF2α	S51	WB	Cell Signaling Technologies	#3398	1:1,000
Fibrillarin		IF	Abcam	#ab166630	1:4,000
G3BP1		WB, IF	Cell Signaling Technologies	#61559	1:1,000
GAPDH		WB	Cell Signaling Technologies	#2118	1:1,000
γ-H2AX	S139	WB, IF	Merck	#05-636	1:1,000
IRF3		WB (Hs)	Abcam	#ab76409	1:1,000
		WB (Mm)	Cell Signaling Technologies	#4302	1:1,000
p-IRF3	S386	WB (Hs)	Abcam	#ab76493	1:1,000
	S396	WB (Mm)	Cell Signaling Technologies	#29047	1:1,000
LGP2		WB (Hs)	Cell Signaling Technologies	#12869	1:1,000
		WB (Mm)	Abcam	#67270	1:1,000
MDA5		WB	Cell Signaling Technologies	#5321	1:1,000
c-PARP1	N214	WB	Cell Signaling Technologies	#9541	1:1,000

Target	Epitope specificity	Application	Manufacturer	Reference	Working dilution
PKR		WB	Cell Signaling Technologies	#12297	1:1,000
		IF	Santa Cruz	#sc-6282	1:500
p-PKR	T446	WB	Abcam	#ab32036	1:1,000
RAD51		IF	Abcam	#ab133534	1:1,000
RIG1		WB	Cell Signaling Technologies	#3743	1:1,000
RNase H1		WB	Santa Cruz	#sc-376326	1:500
RNA:DNA hybrids		IF	Kerafast	#ENH001	1:500
RPA		IF	Abcam	#ab2175	1:1,000
STAT1		WB (Hs)	Cell Signaling Technologies	#9176	1:1,000
		WB (Mm)	Cell Signaling Technologies	#14994	1:1,000
p-STAT1	T701	WB	Cell Signaling Technologies	#9167	1:1,000

Supplementary Table 2. List of siRNAs used in this study.

Target	siRNA type	Species	Reference (Horizon Discovery) or siRNA sequence (5'-3')	Working concentration (nM)
ADAR1	SMARTpool	Hs	L-008630-00-0005	1.25–5
	#1	Hs	J-008630-06-0005	1.25–5
	#2	Hs	J-008630-05-0005	1.25–5
	p150	Hs	GCCUCGCGGGCGCAAUGAA	1.25–5
	3'UTR	Hs	CAGGCUACAGGGUUGUCAC	5
	SMARTpool	Mm	L-063136-01-0005	2.5–10
BRCA1	SMARTpool	Hs	L-003461-00-0005	1.25–5
BRCA2	SMARTpool	Hs	L-003462-00-0005	1.25–5
CGAS	SMARTpool	Hs	L-015607-02-0005	5
IFNAR1	SMARTpool	Hs	L-020209-00-0005	5
LGP2	SMARTpool	Hs	L-010582-00-0005	5
MDA5	SMARTpool	Hs	L-013041-00-0005	5
PKR	SMARTpool	Hs	L-003527-00-0005	5
PLK1	SMARTpool	Hs	L-003290-00-0005	1.25–5
	SMARTpool	Mm	L-040566-00-0005	2.5–10
RIG1	SMARTpool	Hs	L-012511-00-0005	5
CTRL	SMARTpool	Hs, Mm	D-001810-10-05	1.25–5

Supplementary Table 3. List of crRNAs used in this study.

Target	crRNA	Species	Reference (Horizon Discovery)	Working concentration (nM)
ADAR1	#1	Hs	CM-008630-01-0010	12.5
	#2	Hs	CM-008630-02-0010	12.5
	#3	Hs	CM-008630-03-0010	12.5
	#4	Hs	CM-008630-05-0010	12.5
PLK1		Hs	CM-003290-02-0010	12.5
CTRL		Hs	U-007501-01-05	12.5

Supplementary Table 4. List of RT-qPCR probes used in this study.

Target	Probe type	Species	Reference (Thermo Fisher)
CCL5	TaqMan® gene expression assay	H	Hs00982282_m1
Ccl5	TaqMan® gene expression assay	M	Mm01302427_m1
GAPDH	TaqMan® gene expression assay	H	Hs03929097_g1
Gapdh	TaqMan® gene expression assay	M	Mm99999915_g1
Ifi44	TaqMan® gene expression assay	M	Mm00505670_m1
Ifit1	TaqMan® gene expression assay	M	Mm00515153_m1
IFNB1	TaqMan® gene expression assay	H	Hs01077958_s1
Ifnb1	TaqMan® gene expression assay	M	Mm00439552_s1
Mx1	TaqMan® gene expression assay	M	Mm00487796_m1

Supplementary Figures

SUPPLEMENTARY FIGURE 1

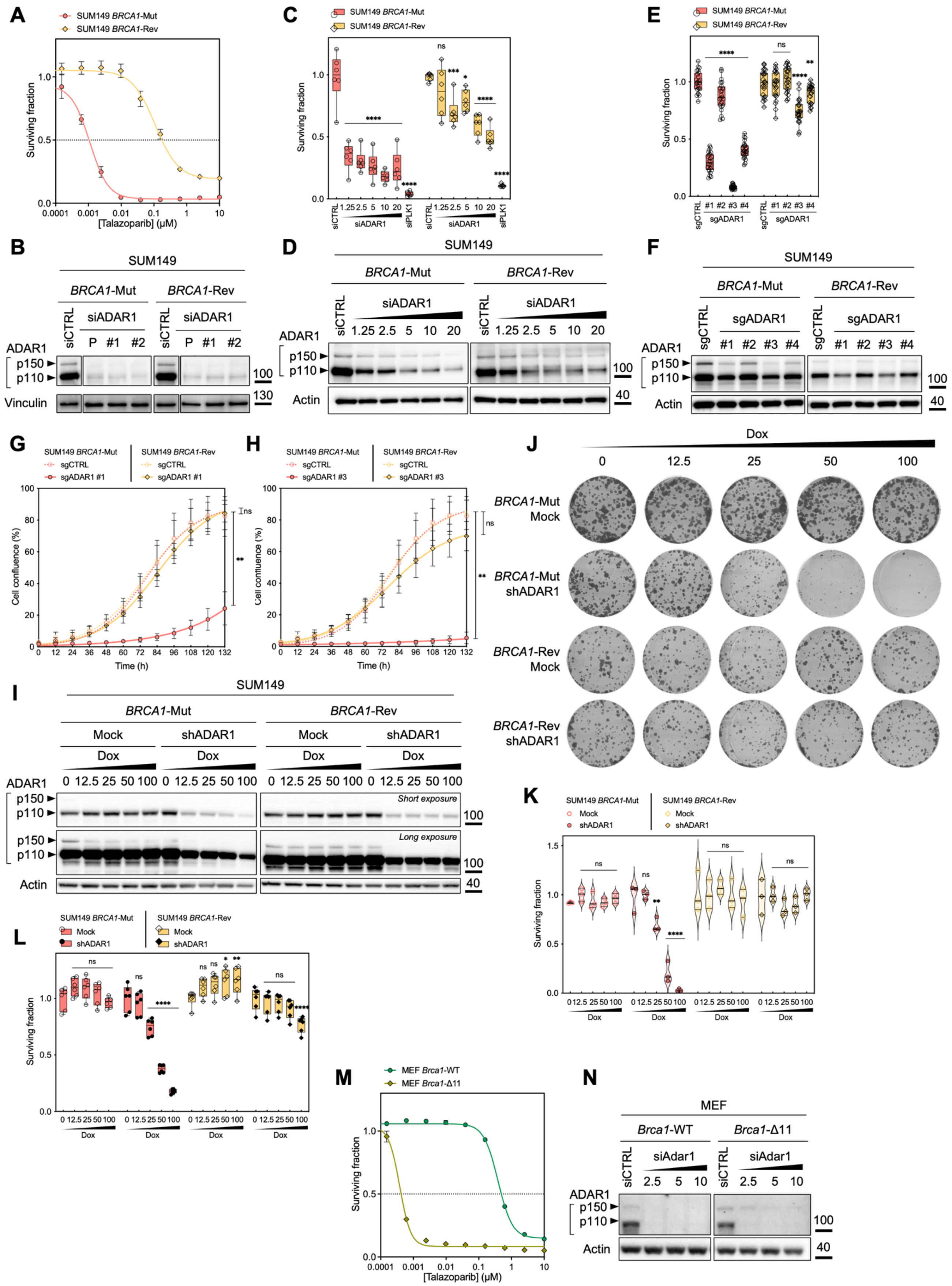

Supplementary Figure 1. Validations of the *BRCA1–ADAR1* synthetic lethal effects in SUM149 and MEF isogenic models.

A. Dose-response survival curves of SUM149 *BRCA1*-Mut and *BRCA1*-Rev cells exposed to increasing concentrations of the PARPi talazoparib for 7 days. Mean ± SD of N=4 values from individual wells, representative of *n*=3 biologically-independent experiments. **B.** Western blot of SUM149 *BRCA1*-Mut and *BRCA1*-Rev cells transfected with *ADAR1* siRNA as described in Fig. 1D, E. Cells were transfected with control, non-targeting siRNA (siCTRL), or with either *ADAR1* siRNA SMARTpool (P) or two individual *ADAR1* siRNAs (#1, #2). 72 hours after transfection, cell lysates were generated and western blotted to detect ADAR1 protein. Data representative of *n*=3 biologically-independent experiments. **C.** Cell survival of SUM149 *BRCA1*-Mut and *BRCA1*-Rev cells transfected with *ADAR1* siRNA in dose-response. Cells were transfected with control, non-targeting siRNA (siCTRL), or with a titration of *ADAR1* siRNA SMARTpool (nM). After transfection, cells were continuously cultured for 7 days, after which cell viability was determined by CellTiter-Glo®. *PLK1*-targeting siRNA (siPLK1) was used as a positive control. Box-and-whiskers indicate median, lower and upper quartiles, and the min to max range; N=6 values from individual wells, representative of *n*=2 biologically-independent experiments, two-way ANOVA post hoc Dunnett's test. **D.** Western blot of SUM149 *BRCA1*-Mut and *BRCA1*-Rev cells transfected with *ADAR1* siRNA as described in C. 72 hours after transfection, cell lysates were generated and western blotted to detect ADAR1 protein. Data representative of *n*=2 biologically-independent experiments. **E.** Cell survival of SUM149 *BRCA1*-Mut and *BRCA1*-Rev cells transfected with *ADAR1* sgRNA as described in Fig.1F, G. Cells were either transfected with control, non-targeting sgRNA (sgCTRL), or with *ADAR1* sgRNAs (#1, #2, #3, #4) in the presence of CRISPR-Cas9 recombinase. After transfection, cells were continuously cultured for 7 days, after which cell viability was determined by CellTiter-Glo®. Box-and-whiskers indicate median, lower and upper quartiles, and the min to max range; N=24 values from individual wells, representative of *n*=3 biologically-independent experiments, two-way ANOVA post hoc Dunnett's test. **F.** Western blot of SUM149 *BRCA1*-Mut and *BRCA1*-Rev cells transfected with *ADAR1* sgRNA as described in Fig. 1F, G. Cells were either transfected with control, non-targeting sgRNA (sgCTRL), or with *ADAR1* sgRNAs (#1, #2, #3, #4) in the presence of CRISPR-Cas9 recombinase. 48 hours after transfection, cell lysates were generated and western blotted to detect ADAR1 protein. Data representative of *n*=3 biologically-independent experiments. **G, H.** Kinetics of proliferation of SUM149 *BRCA1*-Mut and *BRCA1*-Rev cells transfected with two *ADAR1* sgRNA (sgADAR1 #1, G; sgADAR1 #3, H) as described in Fig.1F, G. Cells were either transfected with control, non-targeting sgRNA (sgCTRL), or with *ADAR1* sgRNAs (#1, #3) in the presence of CRISPR-Cas9 recombinase. After transfection, cells were continuously cultured for 6 days, during which cell confluence was measured by use of an Incucyte®. Mean ± SD of N=4 values from individual wells, representative of *n*=3 biologically-independent experiments, two-way ANOVA post hoc Tukey's test. **I.** Western blot of SUM149 *BRCA1*-Mut and *BRCA1*-Rev cells transduced with a

doxycycline-inducible *ADAR1*-targeting shRNA. Cells were either mock-transduced, or transduced with an *ADAR1*-targeting shRNA and subsequently exposed to a titration of doxycycline (ng/mL). 72 hours after addition of doxycycline, cell lysates were generated and western blotted to detect ADAR1 protein. Data representative of *n*=2 biologically-independent experiments. **J, K.** Clonogenic survival of SUM149 *BRCA1*-Mut and *BRCA1*-Rev cells transduced with a doxycycline-inducible *ADAR1*-targeting shRNA as described in I. After addition of doxycycline, cells were continuously cultured for 10 days, after which colonies were stained and counted. Violin plots indicate median, lower and upper quartiles; N=3 values from individual wells, representative of *n*=2 biologically-independent experiments, two-way ANOVA post hoc Dunnett's test. **L.** Cell survival of SUM149 *BRCA1*-Mut and *BRCA1*-Rev cells transduced with a doxycycline-inducible *ADAR1*-targeting shRNA as described in I. After addition of doxycycline, cells were continuously cultured for 7 days, after which cell viability was determined by CellTiter-Glo®. Box-and-whiskers indicate median, lower and upper quartiles, and the min to max range; N=6 values from individual wells, representative of *n*=2 biologically-independent experiments, two-way ANOVA post hoc Dunnett's test. **M.** Dose-response survival curves of MEF *Brca1*-wildtype (WT) and *Brca1*-mutant (Δ11) cells exposed to increasing concentrations of the PARPi talazoparib for 7 days. Mean ± SD of N=9 values from individual wells, representative of *n*=3 biologically-independent experiments. **N.** Western blot of MEF *Brca1*-wildtype (WT) and *Brca1*-mutant (Δ11) cells transfected with *Adar1* siRNA as described in Fig. 2B, C. Cells were transfected with control, non-targeting siRNA (siCTRL), or with a titration of *Adar1* siRNA SMARTpool (nM). 72 hours after transfection, cell lysates were generated and western blotted to detect ADAR1 protein. Data representative of *n*=2 biologically-independent experiments. *P*-values, *<0.05, **<0.01, ***<0.001, ****<0.0001; ns, not significant. Source data are provided as a Source Data file.

SUPPLEMENTARY FIGURE 2

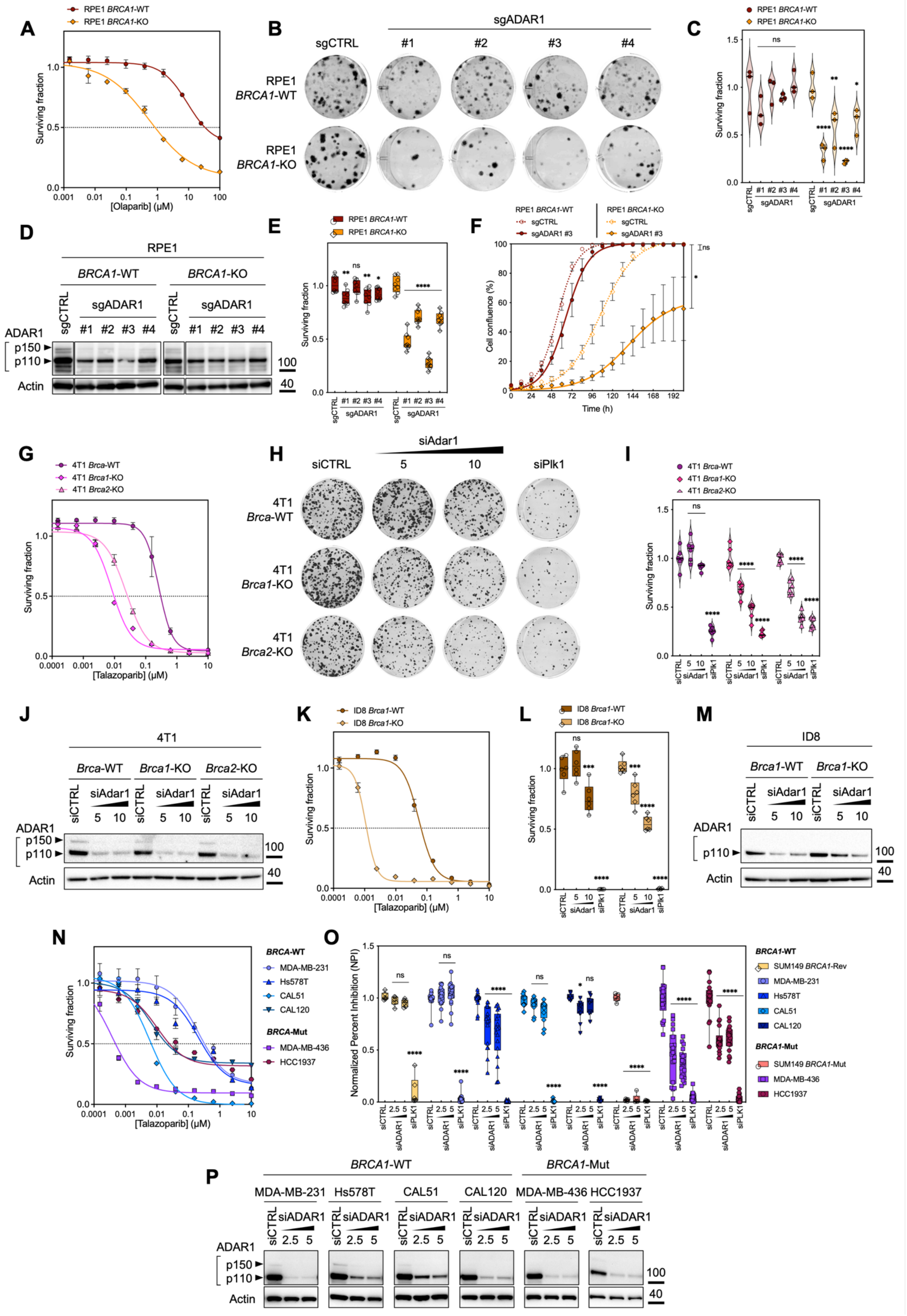

Supplementary Figure 2. Validations of the *BRCA1/2–ADAR1* synthetic lethal effects in RPE1, 4T1, and ID8 isogenic models, and in non-isogenic triple-negative breast cancer cell lines.

A. Dose-response survival curves of RPE1 *BRCA1*-wildtype (WT) and *BRCA1*-knockout (KO) cells exposed to increasing concentrations of the PARPi olaparib for 7 days. Mean ± SD of N=4 values from individual wells, representative of *n*=3 biologically-independent experiments. **B, C.** Clonogenic survival of RPE1 *BRCA1*-wildtype (WT) and *BRCA1*-knockout (KO) cells transfected with *ADAR1* sgRNA. Cells were either transfected with control, non-targeting sgRNA (sgCTRL), or with *ADAR1* sgRNAs (#1, #2, #3, #4) in the presence of CRISPR-Cas9 recombinase. After transfection, cells were continuously cultured for 10 days, after which colonies were stained and counted. Violin plots indicate median, lower and upper quartiles; N=3 values from individual wells, representative of *n*=2 biologically-independent experiments, two-way ANOVA post hoc Dunnett's test. **D.** Western blot of RPE1 *BRCA1*-wildtype (WT) and *BRCA1*-knockout (KO) cells transfected with *ADAR1* sgRNA as described in B, C. 48 hours after transfection, cell lysates were generated and western blotted to detect ADAR1 protein. Data representative of *n*=2 biologically-independent experiments. **E.** Cell survival of RPE1 *BRCA1*-wildtype (WT) and *BRCA1*-knockout (KO) cells transfected with *ADAR1* sgRNA as described in B, C. After transfection, cells were continuously cultured for 7 days, after which cell viability was determined by CellTiter-Glo®. Box-and-whiskers indicate median, lower and upper quartiles, and the min to max range; N=8 values from individual wells, representative of *n*=2 biologically-independent experiments, two-way ANOVA post hoc Dunnett's test. **F.** Kinetics of proliferation of RPE1 *BRCA1*-wildtype (WT) and *BRCA1*-knockout (KO) cells transfected with *ADAR1* sgRNA (sgADAR1 #3) as described in B, C. After transfection, cells were continuously cultured for 9 days, during which cell confluence was measured by use of an Incucyte®. Mean ± SD of N=4 values from individual wells, representative of *n*=2 biologically-independent experiments, two-way ANOVA post hoc Tukey's test. **G.** Dose-response survival curves of 4T1 *Brca*-wildtype (WT), *Brca1*- and *Brca2*-knockout (KO) cells exposed to increasing concentrations of the PARPi talazoparib for 7 days. Mean ± SD of N=4 values from individual wells, representative of *n*=3 biologically-independent experiments. **H, I.** Clonogenic survival of 4T1 *Brca*-wildtype (WT), *Brca1*- and *Brca2*-knockout (KO) cells transfected with *Adar1* siRNA. Cells were transfected with control, non-targeting siRNA (siCTRL), or with a titration of *Adar1* siRNA SMARTpool (nM). After transfection, cells were continuously cultured for 7 days, after which colonies were stained and counted. *PLK1*-targeting siRNA (siPLK1) was used as a positive control. Violin plots indicate median, lower and upper quartiles; N=6 values from individual wells, representative of *n*=2 biologically-independent experiments, two-way ANOVA post hoc Dunnett's test. **J.** Western blot of 4T1 *Brca*-wildtype (WT), *Brca1*- and *Brca2*-knockout (KO) cells transfected with *Adar1* siRNA as described in H, I. 72 hours after transfection, cell lysates were generated and western blotted to detect ADAR1 protein. Data representative of *n*=2 biologically-independent experiments. **K.** Dose-response survival curves of ID8 *Brca1*-wildtype (WT) and *Brca1*-

knockout (KO) cells exposed to increasing concentrations of the PARPi talazoparib for 7 days. Mean ± SD of N=4 values from individual wells, representative of *n*=3 biologically-independent experiments. **L.** Cell survival of ID8 *Brca1*-wildtype (WT) and *Brca1*-knockout (KO) cells transfected with *Adar1* siRNA. Cells were transfected with control, non-targeting siRNA (siCTRL), or with a titration of *Adar1* siRNA SMARTpool (nM). After transfection, cells were continuously cultured for 7 days, after which cell viability was determined by CellTiter-Glo®. *PLK1*-targeting siRNA (siPLK1) was used as a positive control. Box-and-whiskers indicate median, lower and upper quartiles, and the min to max range; N=6 values from individual wells, representative of *n*=2 biologically-independent experiments, two-way ANOVA post hoc Dunnett's test. **M.** Western blot of ID8 *Brca1*-wildtype (WT) and *Brca1*-knockout (KO) cells transfected with *Adar1* siRNA as described in L. 72 hours after transfection, cell lysates were generated and western blotted to detect ADAR1 protein. Data representative of *n*=2 biologically-independent experiments. **N.** Dose-response survival curves of non-isogenic *BRCA1*-wildtype (MDA-MB-231, Hs578T, CAL51, CAL120) and *BRCA1*-mutant (MDA-MB-436, HCC1937) TNBC cells exposed to increasing concentrations of the PARPi talazoparib for 7 days. Mean ± SD of N=4 values from individual wells, representative of *n*=3 biologically-independent experiments. **O.** Cell survival of non-isogenic *BRCA1*-wildtype (MDA-MB-231, Hs578T, CAL51, CAL120) and *BRCA1*-mutant (MDA-MB-436, HCC1937) TNBC cells transfected with *ADAR1* siRNA. Cells were transfected with control, non-targeting siRNA (siCTRL), or with a titration of *ADAR1* siRNA SMARTpool (nM). After transfection, cells were continuously cultured for 7 days, after which cell viability was determined by CellTiter-Glo®. *PLK1*-targeting siRNA (siPLK1) was used as a positive control. Cell survival is presented as normalized percent inhibition (NPI, in which the normalization is carried out relative to both positive and negative controls) to account for the variability in transfection efficiency between cell lines. Box-and-whiskers indicate median, lower and upper quartiles, and the min to max range; N=6–18 values from individual wells, representative of *n*=3 biologically-independent experiments, two-way ANOVA post hoc Dunnett's test. **P.** Western blot of non-isogenic *BRCA1*-wildtype (MDA-MB-231, Hs578T, CAL51, CAL120) and *BRCA1*-mutant (MDA-MB-436, HCC1937) TNBC cells transfected with *ADAR1* siRNA as described in O. 72 hours after transfection, cell lysates were generated and western blotted to detect ADAR1 protein. Data representative of *n*=2 biologically-independent experiments. *P*-values, *<0.05, **<0.01, ***<0.001, ****<0.0001; ns, not significant. Source data are provided as a Source Data file.

SUPPLEMENTARY FIGURE 3

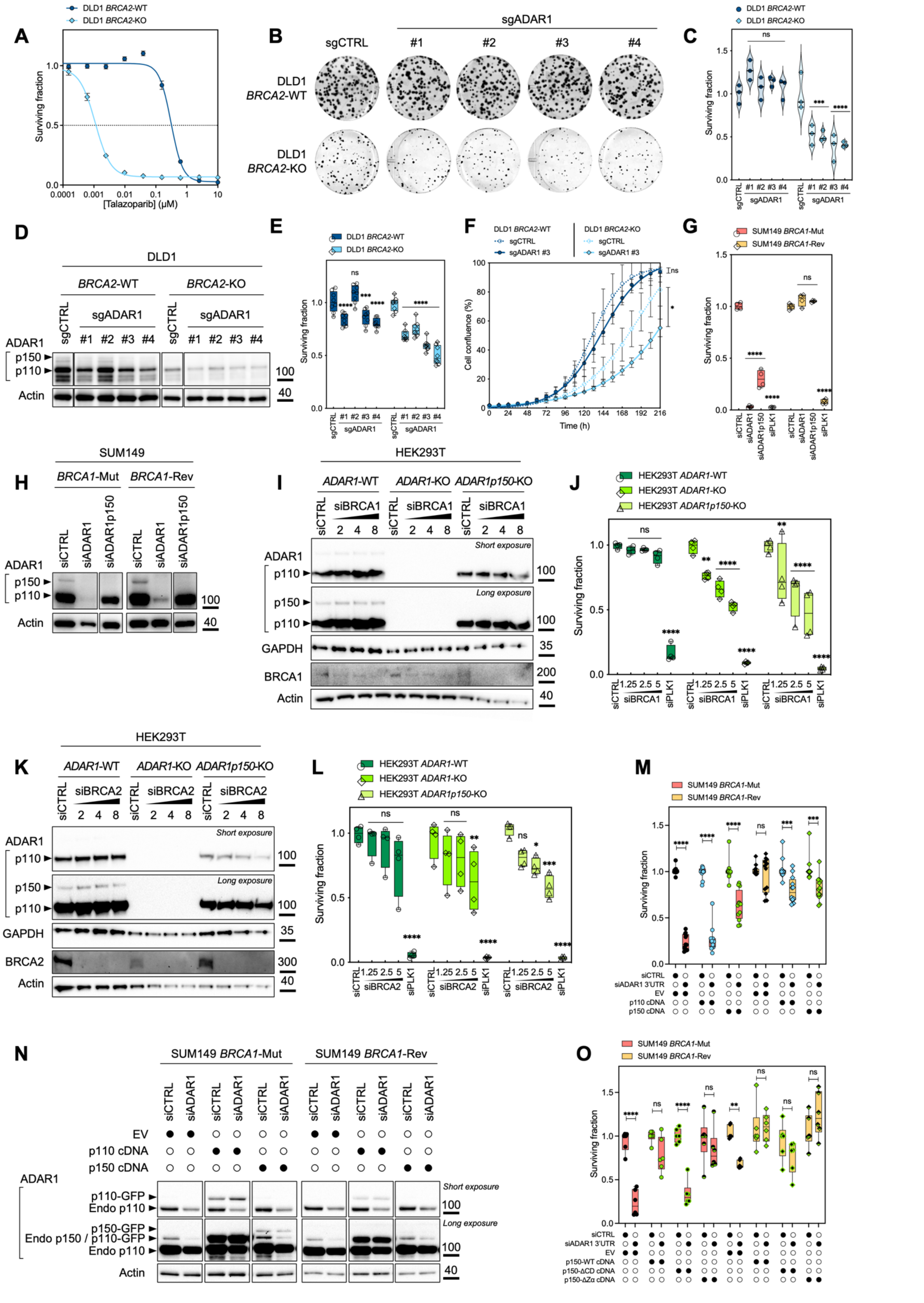

Supplementary Figure 3. Validations of the *BRCA1/2–ADAR1* synthetic lethal effects in DLD1 and HEK293T isogenic models, and assessment of their isoform and functional domain specificity.

A. Dose-response survival curves of DLD1 *BRCA2*-wildtype (WT) and *BRCA2*-knockout (KO) cells exposed to increasing concentrations of the PARPi talazoparib for 7 days. Mean ± SD of N=12 values from individual wells, representative of *n*=3 biologically-independent experiments. **B, C.** Clonogenic survival of DLD1 *BRCA2*-wildtype (WT) and *BRCA2*-knockout (KO) cells transfected with *ADAR1* sgRNA. Cells were either transfected with control, non-targeting sgRNA (sgCTRL), or with *ADAR1* sgRNAs (#1, #2, #3, #4) in the presence of CRISPR-Cas9 recombinase. After transfection, cells were continuously cultured for 10 days, after which colonies were stained and counted. Violin plots indicate median, lower and upper quartiles; N=3 values from individual wells, representative of *n*=2 biologically-independent experiments, two-way ANOVA post hoc Dunnett's test. **D.** Western blot of DLD1 *BRCA2*-wildtype (WT) and *BRCA2*-knockout (KO) cells transfected with *ADAR1* sgRNA as described in B, C. 48 hours after transfection, cell lysates were generated and western blotted to detect ADAR1 protein. Data representative of *n*=2 biologically-independent experiments. **E.** Cell survival of DLD1 *BRCA2*-wildtype (WT) and *BRCA2*-knockout (KO) cells transfected with *ADAR1* sgRNA as described in B, C. After transfection, cells were continuously cultured for 7 days, after which cell viability was determined by CellTiter-Glo®. Box-and-whiskers indicate median, lower and upper quartiles, and the min to max range; N=8 values from individual wells, representative of *n*=2 biologically-independent experiments, two-way ANOVA post hoc Dunnett's test. **F.** Kinetics of proliferation of DLD1 *BRCA2*-wildtype (WT) and *BRCA2*-knockout (KO) cells transfected with *ADAR1* sgRNA (sgADAR1 #3) as described in B, C. After transfection, cells were continuously cultured for 9 days, during which cell confluence was measured by use of an Incucyte®. Mean ± SD of N=4 values from individual wells, representative of *n*=2 biologically-independent experiments, two-way ANOVA post hoc Tukey's test. **G.** Cell survival of SUM149 *BRCA1*-Mut and *BRCA1*-Rev cells transfected with *ADAR1* or ADAR1p150-selective siRNAs. Cells were transfected with control, non-targeting siRNA (siCTRL), or with either *ADAR1* siRNA or ADAR1p150-selective siRNA. After transfection, cells were continuously cultured for 7 days, after which cell viability was determined by CellTiter-Glo®. *PLK1*-targeting siRNA (siPLK1) was used as a positive control. Box-and-whiskers indicate median, lower and upper quartiles, and the min to max range; N=4 values from individual wells, representative of *n*=2 biologically-independent experiments, two-way ANOVA post hoc Dunnett's test. **H.** Western blot of SUM149 *BRCA1*-Mut and *BRCA1*-Rev cells transfected with *ADAR1* or ADAR1p150-selective siRNAs, as described in G. 72 hours after transfection, cell lysates were generated and western blotted to detect ADAR1 protein isoforms. Data representative of *n*=2 biologically-independent experiments. **I.** Western blot of HEK293T *ADAR1*-wildtype (WT), *ADAR1*- and *ADAR1p150*-knockout (KO) cells transfected with *BRCA1* siRNA. Cells were transfected with control, non-targeting siRNA (siCTRL) or with a titration of *BRCA1* siRNA SMARTpool (nM). 72 hours after

transfection, cell lysates were generated and western blotted to detect ADAR1 and BRCA1 proteins. Data representative of *n*=3 biologically-independent experiments. Short and long exposure times were used to image some membranes. **J.** Cell survival of HEK293T *ADAR1*-wildtype (WT), *ADAR1*- and *ADAR1p150*-knockout (KO) cells transfected with *BRCA1* siRNA, as described in **I**. After transfection, cells were continuously cultured for 6 days, after which cell viability was determined by CellTiter-Glo®. *PLK1*-targeting siRNA (siPLK1) was used as a positive control. Box-and-whiskers indicate median, lower and upper quartiles, and the min to max range; N=4 values from individual wells, representative of *n*=3 biologically-independent experiments, two-way ANOVA post hoc Dunnett's test. **K.** Western blot of HEK293T *ADAR1*-wildtype (WT), *ADAR1*- and *ADAR1p150*-knockout (KO) cells transfected with *BRCA2* siRNA. Cells were transfected with control, non-targeting siRNA (siCTRL) or with a titration of *BRCA2* siRNA SMARTpool (nM). 72 hours after transfection, cell lysates were generated and western blotted to detect ADAR1 and BRCA2 proteins. Data representative of *n*=3 biologically-independent experiments. Short and long exposure times were used to image some membranes. **L.** Cell survival of HEK293T *ADAR1*-wildtype (WT), *ADAR1*- and *ADAR1p150*-knockout (KO) cells transfected with *BRCA2* siRNA, as described in K. After transfection, cells were continuously cultured for 6 days, after which cell viability was determined by CellTiter-Glo®. *PLK1*-targeting siRNA (siPLK1) was used as a positive control. Box-and-whiskers indicate median, lower and upper quartiles, and the min to max range; N=4 values from individual wells, representative of *n*=3 biologically-independent experiments, two-way ANOVA post hoc Dunnett's test. **M.** Cell survival of SUM149 *BRCA1*-Mut and *BRCA1*-Rev cells transfected with *ADAR1* siRNA in the context of exogenous overexpression of wildtype ADAR1p110 or ADAR1p150. Cells were transfected with control, non-targeting siRNA (siCTRL) or with an individual siRNA targeting *ADAR1* 3'UTR (5 nM), and with plasmid constructs containing no cDNA (empty vector, EV), ADAR1p110 or ADAR1p150 cDNA (100 pg/mL). After transfection, cells were continuously cultured for 7 days, after which cell viability was determined by CellTiter-Glo®. Box-and-whiskers indicate median, lower and upper quartiles, and the min to max range; N=12 values from individual wells, representative of *n*=2 biologically-independent experiments, two-way ANOVA post hoc Šídák's test. **N.** Western blot of SUM149 *BRCA1*-Mut and *BRCA1*-Rev cells transfected with *ADAR1* siRNA in the context of exogenous overexpression of wildtype ADAR1p110 or ADAR1p150. Cells were transfected with control, non-targeting siRNA (siCTRL) or with an individual siRNA targeting *ADAR1* 3'UTR (5 nM), and with plasmid constructs containing no cDNA (empty vector, EV), ADAR1p110 or ADAR1p150 cDNA (100 pg/mL). 72 hours after transfection, cell lysates were generated and western blotted to detect ADAR1 protein. Data representative of *n*=2 biologically-independent experiments. Short and long exposure times were used to image some membranes. **O.** Cell survival of SUM149 *BRCA1*-Mut and *BRCA1*-Rev cells transfected with *ADAR1* siRNA in the context of exogenous overexpression of wildtype and mutant ADAR1p150. Cells were transfected with control, non-targeting siRNA (siCTRL) or with an individual siRNA targeting *ADAR1* 3'UTR (5 nM), and with plasmid constructs containing no cDNA (empty vector, EV), wildtype, catalytically-inactive (ΔCD) or Zα

domain-mutated (ΔZα) ADAR1p150 cDNA (100 pg/mL). After transfection, cells were continuously cultured for 7 days, after which cell viability was determined by CellTiter-Glo®. Box-and-whiskers indicate median, lower and upper quartiles, and the min to max range; N=6 values from individual wells, representative of *n*=2 biologically-independent experiments, two-way ANOVA post hoc Šídák's test. *P*-values*,* *<0.05, **<0.01, ***<0.001, ****<0.0001; ns, not significant. Source data are provided as a Source Data file.

SUPPLEMENTARY FIGURE 4

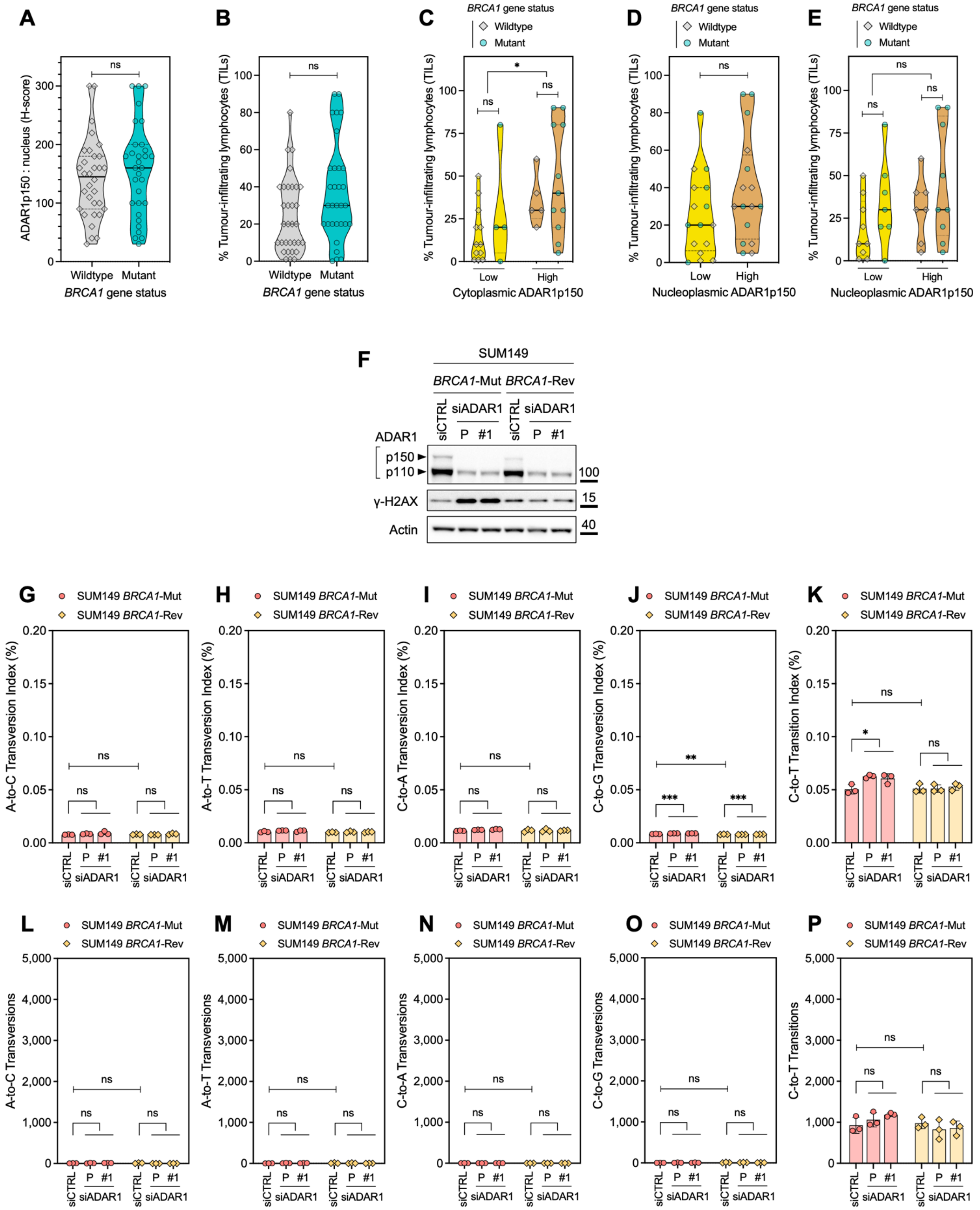

Supplementary Figure 4. ADAR1p150 expression and levels of tumor-infiltrating lymphocytes in *BRCA1*-mutant and -wildtype triple-negative breast cancer tumors.

A. Pathological evaluation of nucleoplasmic expression of ADAR1p150 according to *BRCA1* gene status (*BRCA1*-wildtype vs. *BRCA1*-mutant) in a cohort of 63 treatment-naïve triple-negative breast cancer (TNBC) patients. ADAR1p150 expression is displayed as a H-score (range, 0–300). Violin plots indicate median, lower and upper quartiles; N=32 [*BRCA1*-wildtype], N=31 [*BRCA1*-mutant] values from individual tumor samples, two-tailed Mann-Whitney U test. **B.** Percentage of tumor-infiltrating lymphocytes (TILs) in TNBC tumors from the cohort described in A, according to *BRCA1* gene status (*BRCA1*-wildtype vs. *BRCA1*-mutant). Violin plots indicate median, lower and upper quartiles; N=32 [*BRCA1*-wildtype], N=31 [*BRCA1*-mutant] values from individual tumor samples, two-tailed Mann-Whitney U test. **C.** Percentage of TILs in TNBC tumors from the cohort described in A, according to *BRCA1* gene status (*BRCA1*-wildtype vs. *BRCA1*-mutant) and cytoplasmic ADAR1p150 expression (based on Fig. 3A; ADAR1p150-low, lower quartile of H-score; ADAR1p150-high, upper quartile of H-score). Violin plots indicate median, lower and upper quartiles; N=17 [*BRCA1*-wildtype], N=15 [*BRCA1*-mutant] values from individual tumor samples, two-way ANOVA post hoc Šidák's test. **D.** Percentage of TILs in TNBC tumors from the cohort described in A, according to nucleoplasmic ADAR1p150 expression (based on A; ADAR1p150-low, lower quartile of H-score; ADAR1p150-high, upper quartile of H-score). Violin plots indicate median, lower and upper quartiles; N=16 [*BRCA1*-wildtype], N=16 [*BRCA1*-mutant] values from individual tumor samples, two-tailed Mann-Whitney U test. **E.** Percentage of TILs in TNBC tumors from the cohort described in A, according to *BRCA1* gene status (*BRCA1*-wildtype vs. *BRCA1*-mutant) and nucleoplasmic ADAR1p150 expression (based on A; ADAR1p150-low, lower quartile of H-score; ADAR1p150-high, upper quartile of H-score). Violin plots indicate median, lower and upper quartiles; N=16 [*BRCA1*-wildtype], N=16 [*BRCA1*-mutant] values from individual tumor samples, two-way ANOVA post hoc Šidák's test. **F.** Western blot of SUM149 *BRCA1*-Mut and *BRCA1*-Rev cells transfected with *ADAR1* siRNA as described in Fig. 3F, G. Cells were transfected with control, non-targeting siRNA (siCTRL), or with either *ADAR1* siRNA SMARTpool (P) or an individual *ADAR1* siRNA (#1). 72 hours after transfection, cell lysates were generated and western blotted to detect ADAR1 and γ-H2AX proteins. Data representative of *n*=3 biologically-independent experiments. **G-P.** RNA editing levels of all possible types of editing displayed as RNA editing index (G-K) or number of RNA editing sites (L-P) in SUM149 *BRCA1*-Mut and *BRCA1*-Rev cells transfected with *ADAR1* siRNA as described in Fig. 3F, G. Cells were transfected with control, non-targeting siRNA (siCTRL), or with either *ADAR1* siRNA SMARTpool (P) or an individual *ADAR1* siRNA (#1). 60 hours after transfection, RNA extracts were generated and subjected to RNA-Seq. Bar plots indicate mean ± SD; *n*=3 biological replicates, two-way ANOVA post hoc Tukey's test. *P*-values, *<0.05, **<0.01, ***<0.001, ****<0.0001; ns, not significant. Source data are provided as a Source Data file.

SUPPLEMENTARY FIGURE 5

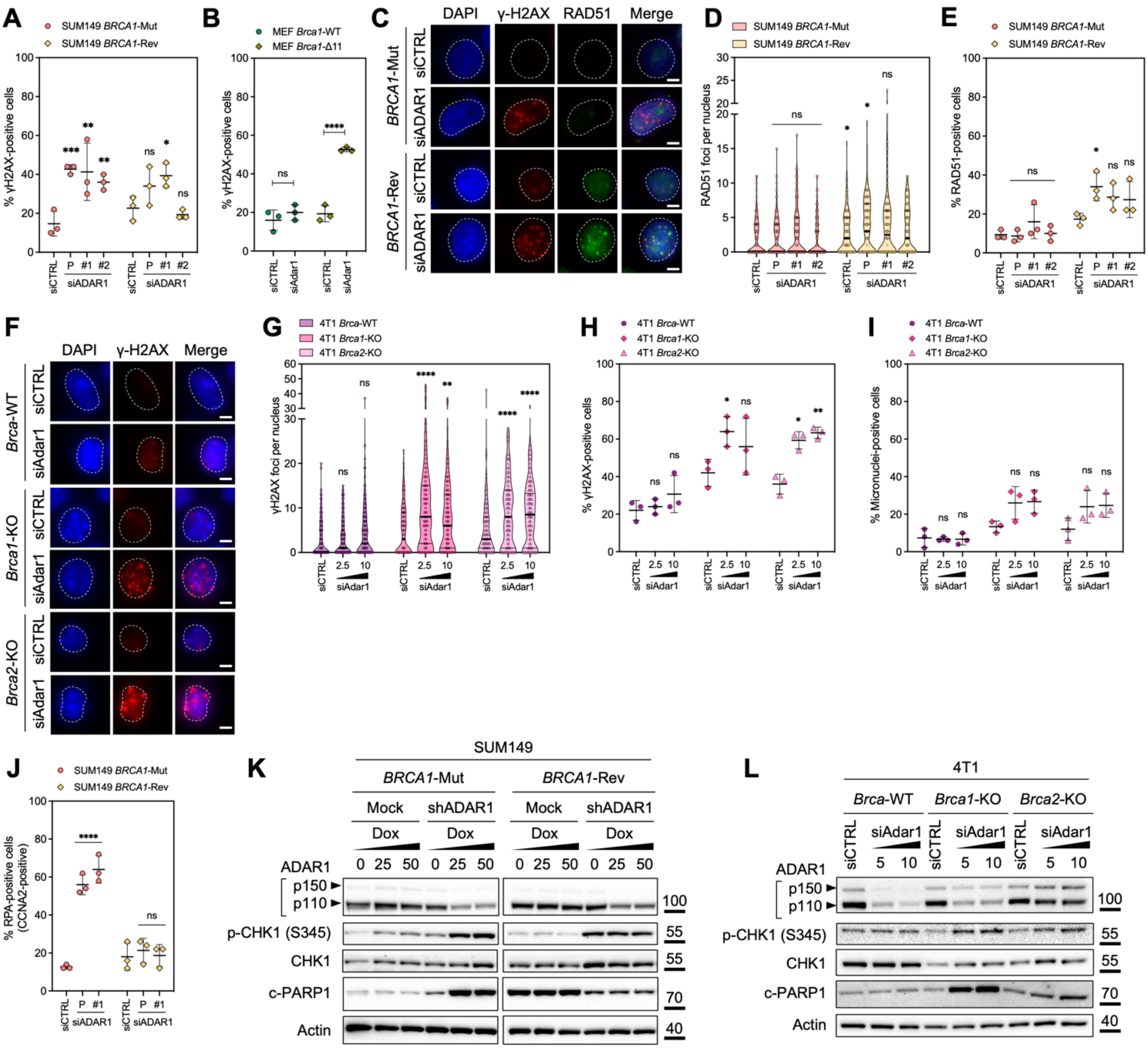

Supplementary Figure 5. Characterization of the DNA damage response associated with ADAR1 loss in *BRCA1/2*-isogenic systems.

A, B. Quantifications of γ-H2AX foci in SUM149 *BRCA1*-Mut and *BRCA1*-Rev cells (A) or MEF *Brca1*-wildtype (WT) and *Brca1*-mutant (Δ11) cells (B) transfected with *ADAR1* siRNA as described in Fig. 4A-D. Cells were transfected with control, non-targeting siRNA (siCTRL), or with either *ADAR1* siRNA SMARTpool (P) or two individual *ADAR1* siRNAs (#1, #2). 72 hours after transfection, cells were fixed and stained with DAPI and γ-H2AX immunostaining. Data are presented as the percentage of γ-H2AX-positive cells (cells harboring >5 nuclear γ-H2AX foci) in the assessed population. Scatter dot plots indicate mean ± SD; N=3 values from individual microscopic fields, representative of *n*=3 biologically-independent experiments, two-way ANOVA post hoc Dunnett's test (A) or post hoc Šidák's test (B). **C-E.** Representative immunofluorescence images (C) and associated quantifications (D, E) of RAD51 foci in SUM149 *BRCA1*-Mut and *BRCA1*-Rev cells transfected with *ADAR1* siRNA as described in Fig. 4A, B. Cells were transfected with control, non-targeting siRNA (siCTRL), or with either *ADAR1* siRNA SMARTpool (P) or two individual *ADAR1* siRNAs (#1, #2). 72 hours after transfection, cells were fixed and stained with DAPI and RAD51 immunostaining. Data are presented as the number of RAD51 foci per nucleus (D) or the percentage of RAD51-positive cells (cells harboring >5 nuclear RAD51 foci) in the assessed population (E). Violin plots indicate median, lower and upper quartiles (D); scatter dot plots indicate mean ± SD (E); N=150 values from individual nuclei (D) or N=3 values from individual microscopic fields (E), representative of *n*=3 biologically-independent experiments, two-way ANOVA post hoc Dunnett's test. **F-H.** Representative immunofluorescence images (F) and associated quantifications (G, H) of γ-H2AX foci in 4T1 *Brca*-wildtype (WT), *Brca1*- and *Brca2*-knockout (KO) cells transfected with *Adar1* siRNA. Cells were transfected with control, non-targeting siRNA (siCTRL), or with a titration of *Adar1* siRNA SMARTpool (nM). 72 hours after transfection, cells were fixed and stained with DAPI and γ-H2AX immunostaining. Data are presented as the number of γ-H2AX foci per nucleus (G) or the percentage of γ-H2AX-positive cells (cells harboring >5 nuclear γ-H2AX foci) in the assessed population (H). Violin plots indicate median, lower and upper quartiles (G); scatter dot plots indicate mean ± SD (H); N=150 values from individual nuclei (G) or N=3 values from individual microscopic fields (H), representative of *n*=2 biologically-independent experiments, two-way ANOVA post hoc Šidák's test. **I.** Quantifications of micronuclei in 4T1 *Brca*-wildtype (WT), *Brca1*- and *Brca2*-knockout (KO) cells transfected with *Adar1* siRNA as described in F-H. 72 hours after transfection, cells were fixed and stained with DAPI. Data are presented as the percentage of micronuclei-positive cells (cells harboring >1 micronucleus) in the assessed population. Scatter dot plots indicate mean ± SD; N=3 values from individual microscopic fields, representative of *n*=2 biologically-independent experiments, two-way ANOVA post hoc Šidák's test. **J.** Quantifications of RPA foci in CCNA2-positive SUM149 *BRCA1*-Mut and *BRCA1*-Rev cells transfected with *ADAR1* siRNA as described in Fig. 4I, J. Cells were transfected with control, non-targeting siRNA

(siCTRL), or with either *ADAR1* siRNA SMARTpool (P) or an individual *ADAR1* siRNAs (#1). 72 hours after transfection, cells were fixed and stained with DAPI, RPA and CCNA2 immunostainings. Data are presented as the percentage of RPA-positive cells (cells harboring >5 nuclear RPA foci) in the assessed population. Scatter dot plots indicate mean ± SD; N=3 values from individual microscopic fields, representative of *n*=3 biologically-independent experiments, two-way ANOVA post hoc Dunnett's test. **K.** Western blot of SUM149 *BRCA1*-Mut and *BRCA1*-Rev cells transduced with a doxycycline-inducible *ADAR1*-targeting shRNA. Cells were either mock-transduced, or transduced with an *ADAR1*-targeting shRNA and subsequently exposed to a titration of doxycycline (ng/mL) for 72 hours. After this, cell lysates were generated and western blotted to detect phosphorylated CHK1 (p-CHK1), CHK1 and cleaved-PARP1 (c-PARP1) proteins. Data representative of *n*=2 biologically-independent experiments. Appropriate silencing of ADAR1 was verified as shown in Fig. 6A; for the sake of clarity, the ADAR1 and actin blots were duplicated from Fig. 6A. **L.** Western blot of 4T1 *Brca*-wildtype (WT), *Brca1*- and *Brca2*-knockout (KO) cells transfected with *Adar1* siRNA as described in F-H. 72 hours after transfection, cell lysates were generated and western blotted to detect phosphorylated CHK1 (p-CHK1), CHK1 and cleaved-PARP1 (c-PARP1) proteins. Data representative of *n*=2 biologically-independent experiments. *P*-values, *<0.05, **<0.01, ***<0.001, ****<0.0001; ns, not significant. Source data are provided as a Source Data file.

SUPPLEMENTARY FIGURE 6

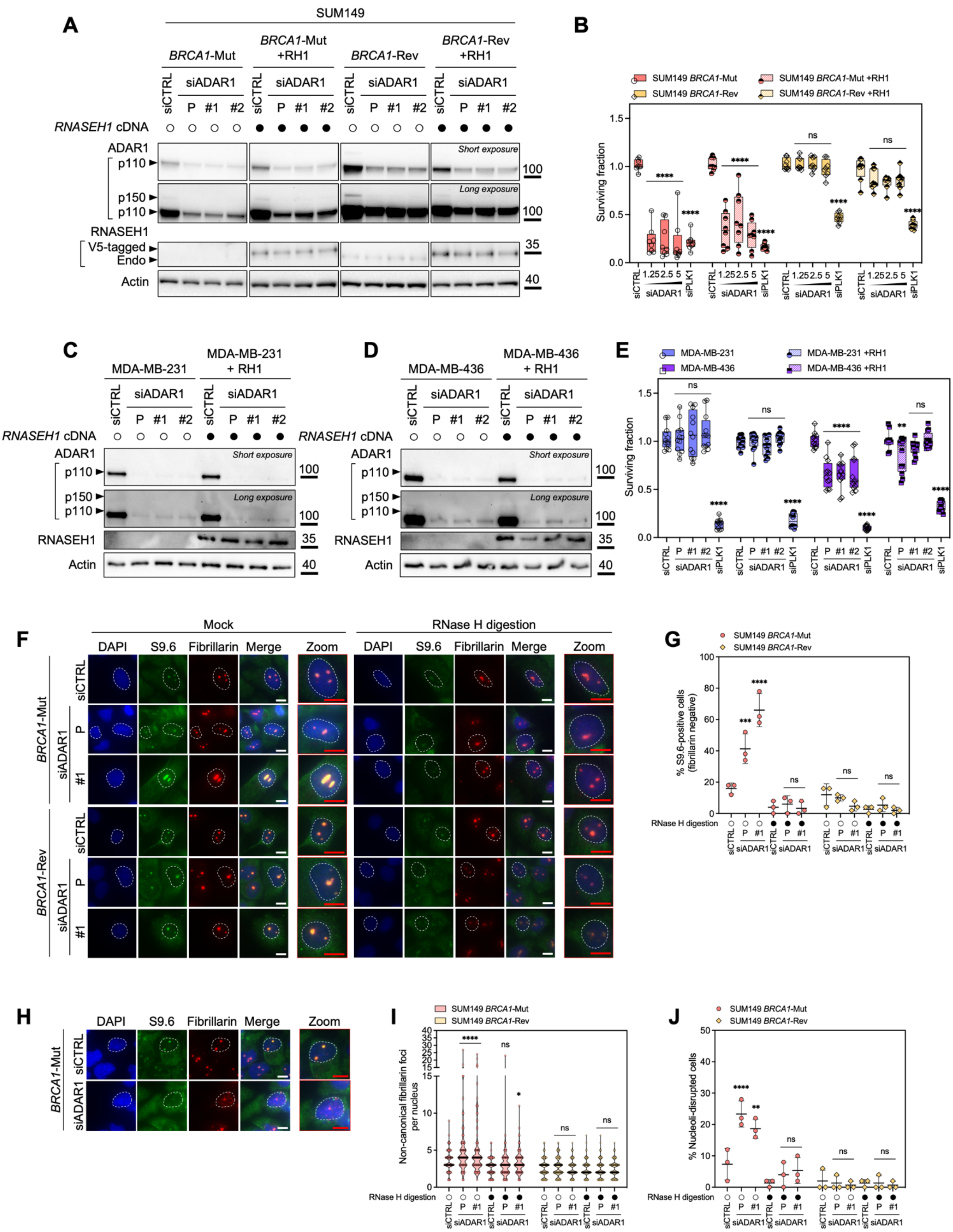

Supplementary Figure 6. Role of R-loops in the *BRCA1–ADAR1* synthetic lethal effects.

A. Western blot of SUM149 *BRCA1*-Mut and *BRCA1*-Rev cells transfected with *ADAR1* siRNA in the context of exogenous overexpression of RNase H1 (RH1). Cells were transduced with a plasmid construct containing *RNASEH1* cDNA to induce stable overexpression of RNase H1, and were transfected with control, non-targeting siRNA (siCTRL), or with either *ADAR1* siRNA SMARTpool (P) or two individual *ADAR1* siRNAs (#1, #2). 72 hours after transfection, cell lysates were generated and western blotted to detect ADAR1 and RNase H1 proteins. Data representative of *n*=3 biologically-independent experiments. Short and long exposure times were used to image some membranes. **B.** Cell survival of SUM149 *BRCA1*-Mut and *BRCA1*-Rev cells transfected with *ADAR1* siRNA in the context of exogenous overexpression of RNase H1 (RH1), as described in A. After transfection, cells were continuously cultured for 7 days, after which cell viability was determined by CellTiter-Glo®. *PLK1*-targeting siRNA (siPLK1) was used as a positive control. Box-and-whiskers indicate median, lower and upper quartiles, and the min to max range; N=8 values from individual wells, representative of *n*=3 biologically-independent experiments, two-way ANOVA post hoc Dunnett's test. **C, D.** Western blots of MDA-MB-231 (C) and MDA-MB-436 (D) cells transfected with *ADAR1* siRNA in the context of exogenous overexpression of RNase H1 (RH1). Cells were transduced with a plasmid construct containing *RNASEH1* cDNA to induce stable overexpression of RNase H1, and were transfected with control, non-targeting siRNA (siCTRL), or with either *ADAR1* siRNA SMARTpool (P) or two individual *ADAR1* siRNAs (#1, #2). 72 hours after transfection, cell lysates were generated and western blotted to detect ADAR1 and RNase H1 proteins. Data representative of *n*=3 biologically-independent experiments. Short and long exposure times were used to image some membranes. **E.** Cell survival of MDA-MB-231 and MDA-MB-436 cells transfected with *ADAR1* siRNA in the context of exogenous overexpression of RNase H1 (RH1), as described in C, D. After transfection, cells were continuously cultured for 7 days, after which cell viability was determined by CellTiter-Glo®. *PLK1*-targeting siRNA (siPLK1) was used as a positive control. Box-and-whiskers indicate median, lower and upper quartiles, and the min to max range; N=12 values from individual wells, representative of *n*=3 biologically-independent experiments, two-way ANOVA post hoc Dunnett's test. **F, G.** Representative immunofluorescence images (F) and associated quantifications (G) of R-loops in SUM149 *BRCA1*-Mut and *BRCA1*-Rev cells transfected with *ADAR1* siRNA as described in Fig. 5C, D. Cells were transfected with control, non-targeting siRNA (siCTRL), or with either *ADAR1* siRNA SMARTpool (P) or an individual *ADAR1* siRNA (#1). 72 hours after transfection, cells were fixed and stained with DAPI, S9.6 and fibrillarin immunostainings. Data are presented as the percentage of S9.6-positive cells (cells harboring >5 nuclear S9.6 foci, outside nucleoli) in the assessed population. Scatter dot plots indicate mean ± SD; N=3 values from individual microscopic fields, representative of *n*=3 biologically-independent experiments, two-way ANOVA post hoc Tukey's test. **H-J.** Representative immunofluorescence images (H) and associated

quantifications (I, J) of aberrantly-shaped or ectopic nucleoli in SUM149 *BRCA1*-Mut and *BRCA1*-Rev cells transfected with *ADAR1* siRNA as described in Fig. 5C, D. Cells were transfected with control, non-targeting siRNA (siCTRL), or with either *ADAR1* siRNA SMARTpool (P) or an individual *ADAR1* siRNA (#1). 72 hours after transfection, cells were fixed and stained with DAPI, S9.6 and fibrillarin immunostainings. Data are presented as the number of non-canonical fibrillarin foci per nucleus (I) or the percentage of nucleoli-disrupted cells (cells harboring >5 nuclear non-canonical fibrillarin foci) in the assessed population (J). Violin plots indicate median, lower and upper quartiles (I); scatter dot plots indicate mean ± SD (J); N=150 values from individual nuclei (I) or N=3 values from individual microscopic fields (J), representative of *n*=3 biologically-independent experiments, two-way ANOVA post hoc Tukey's test. *P*-values, *<0.05, **<0.01, ***<0.001, ****<0.0001; ns, not significant. Source data are provided as a Source Data file.

SUPPLEMENTARY FIGURE 7

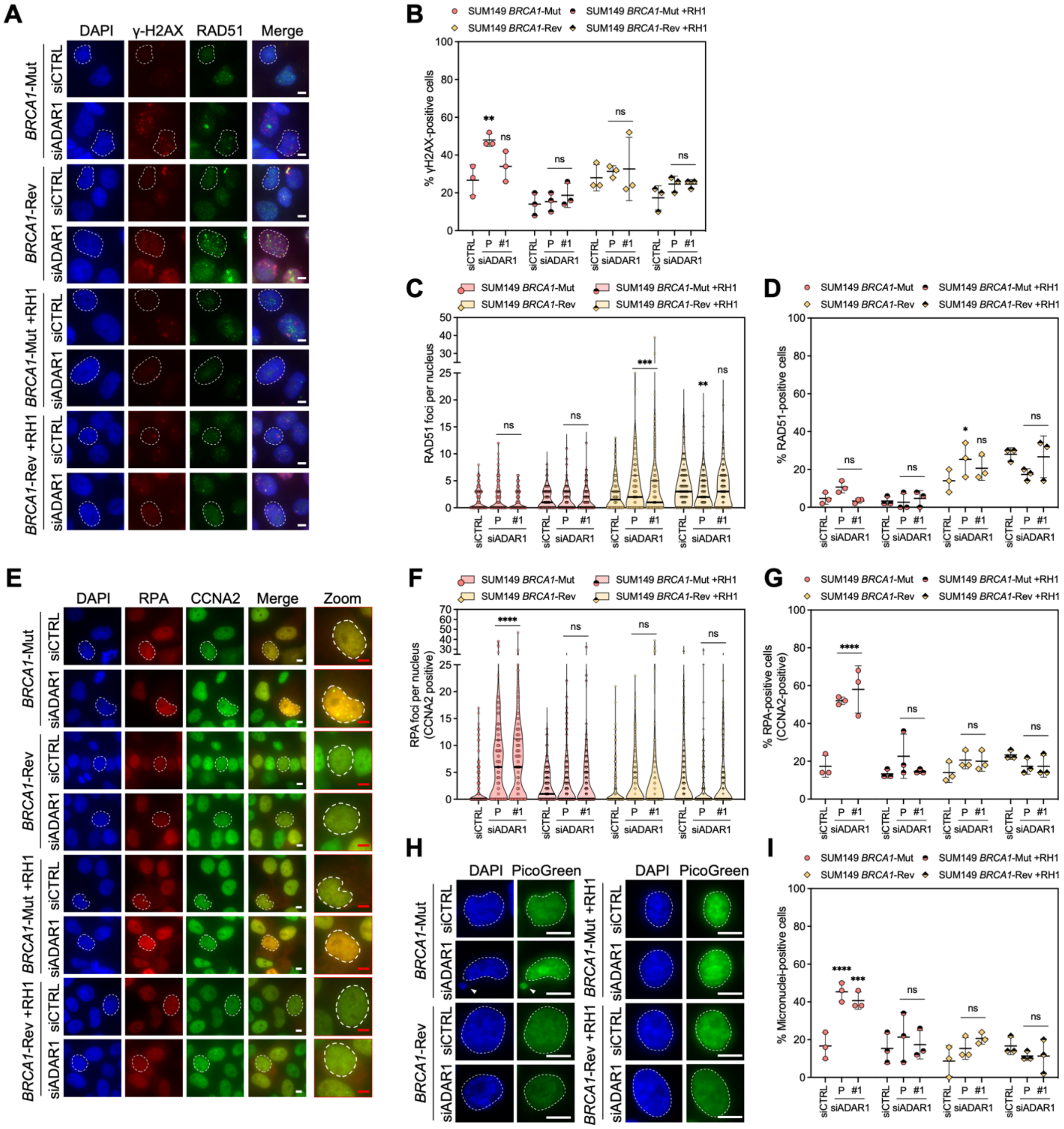

Supplementary Figure 7. Role of R-loops in the DNA damage response associated with the *BRCA1–ADAR1* synthetic lethal effects.

A-D. Representative immunofluorescence images (A) and associated quantifications of γ-H2AX foci (B) and RAD51 foci (C, D) in SUM149 *BRCA1*-Mut and *BRCA1*-Rev cells transfected with *ADAR1* siRNA in the context of exogenous overexpression of RNase H1 (RH1). Cells were transduced with a plasmid construct containing *RNASEH1* cDNA to induce stable overexpression of RNase H1, and were transfected with control, non-targeting siRNA (siCTRL), or with either *ADAR1* siRNA SMARTpool (P) or an individual *ADAR1* siRNAs (#1). 72 hours after transfection, cells were fixed and stained with DAPI, γ-H2AX and RAD51 immunostainings. Data are presented as the percentage of positive cells (cells harboring >5 nuclear γ-H2AX or RAD51 foci) in the assessed population (B, D), or the number of RAD51 foci per nucleus (C). Scatter dot plots indicate mean ± SD (B, D); violin plots indicate median, lower and upper quartiles (C); N=150 values from individual nuclei (C) or N=3 values from individual microscopic fields (B, D), representative of *n*=2 biologically-independent experiments, two-way ANOVA post hoc Dunnett's test. **E-G.** Representative immunofluorescence images (E) and associated quantifications (F, G) of RPA foci in CCNA2-positive SUM149 *BRCA1*-Mut and *BRCA1*-Rev cells transfected with *ADAR1* siRNA in the context of exogenous overexpression of RNase H1 (RH1), as described in A-D. 72 hours after transfection, cells were fixed and stained with DAPI, RPA and CCNA2 immunostainings. Data are presented as the number of RPA foci per nucleus (F), or the percentage of RPA-positive cells (cells harboring >5 nuclear RPA foci) in the assessed population (G). Violin plots indicate median, lower and upper quartiles (F); scatter dot plots indicate mean ± SD (G); N=150 values from individual nuclei (F) or N=3 values from individual microscopic fields (G), representative of *n*=2 biologically-independent experiments, two-way ANOVA post hoc Dunnett's test. **H, I.** Representative immunofluorescence images (H) and associated quantifications (I) of micronuclei in SUM149 *BRCA1*-Mut and *BRCA1*-Rev cells transfected with *ADAR1* siRNA in the context of exogenous overexpression of RNase H1 (RH1), as described in A-D. 72 hours after transfection, cells were fixed and stained with DAPI and PicoGreen®. Data are presented as the percentage of micronuclei-positive cells (cells harboring >1 micronucleus) in the assessed population. Scatter dot plots indicate mean ± SD; N=3 values from individual microscopic fields, representative of *n*=2 biologically-independent experiments, two-way ANOVA post hoc Dunnett's test. *P*-values, *<0.05, **<0.01, ***<0.001, ****<0.0001; ns, not significant. Source data are provided as a Source Data file.

SUPPLEMENTARY FIGURE 8

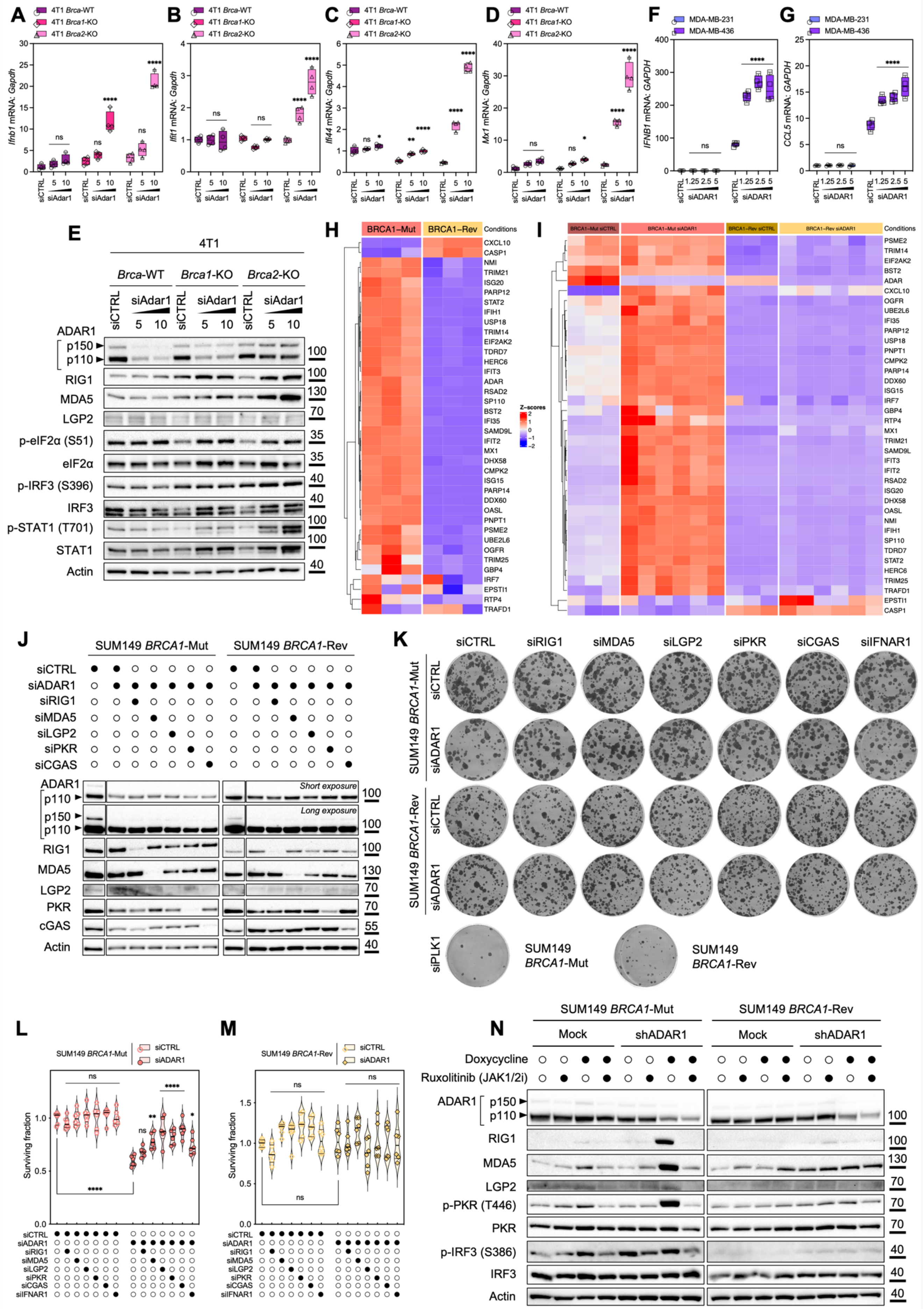

Supplementary Figure 8. Type I interferon response and the role of pattern recognition receptors in the *BRCA1–ADAR1* synthetic lethal effects.

A-D. RT-qPCR of *Ifnb1* (A), *Ifit1* (B), *Ifi44* (C) and *Mx1* (D) mRNAs in 4T1 *Brca*-wildtype (WT), *Brca1*- and *Brca2*-knockout (KO) cells transfected with *Adar1* siRNA. Cells were transfected with control, non-targeting siRNA (siCTRL), or with a titration of *Adar1* siRNA SMARTpool (nM). 72 hours after transfection, RNA extracts were generated and subjected to RT-qPCR. *Ifnb1*, *Ifit1*, *Ifi44* and *Mx1* mRNAs were analyzed separately relative to *Gapdh*. Box-and-whiskers show arbitrary units of gene expression normalized to the *Brca*-wildtype (WT) siCTRL condition, and indicate median, lower and upper quartiles, and the min to max range; N=4 values from individual measurements, representative of *n*=2 biologically-independent experiments, two-way ANOVA post hoc Dunnett's test. **E.** Western blot of 4T1 *Brca*-wildtype (WT), *Brca1*- and *Brca2*-knockout (KO) cells transfected with *Adar1* siRNA, as described in A-D. 72 hours after transfection, cell lysates were generated and western blotted to detect RIG1, MDA5, LGP2, phosphorylated eIF2α (p-eIF2α), eIF2α, phosphorylated IRF3 (p-IRF3), IRF3, phosphorylated STAT1 (p-STAT1) and STAT1 proteins. Data representative of *n*=2 biologically-independent experiments. Appropriate silencing of ADAR1 was verified as shown in Supplementary Fig. 5L; for the sake of clarity, the ADAR1 and actin blots were duplicated from Supplementary Fig. 5L. **F, G.** RT-qPCR of *IFNB1* (F) and *CCL5* (G) mRNAs in MDA-MB-231 and MDA-MB-436 cells transfected with *ADAR1* siRNA. Cells were either transfected with control, non-targeting siRNA (siCTRL) or with a titration of *ADAR1* siRNA SMARTpool (nM). 72 hours after transfection, RNA extracts were generated and subjected to RT-qPCR. *IFNB1* and *CCL5* mRNAs were analyzed separately relative to *GAPDH*. Box-and-whiskers show arbitrary units of gene expression normalized to the MDA-MB-231 siCTRL condition, and indicate median, lower and upper quartiles, and the min to max range; N=4 values from individual measurements, representative of *n*=2 biologically-independent experiments, two-way ANOVA post hoc Dunnett's test. **H.** Heatmap showing transcript-level changes of genes within the interferon-stimulated genes (ISG) signature in SUM149 *BRCA1*-Mut and *BRCA1*-Rev cells. Color mapping indicates z-scores of gene expression based on RNA-Seq. *n*=3 biological replicates. **I**. Heatmap showing transcript-level changes of genes within the interferon-stimulated genes (ISG) signature in SUM149 *BRCA1*-Mut and *BRCA1*-Rev cells transfected with *ADAR1* siRNA. Cells were transfected with control, non-targeting siRNA (siCTRL), or with *ADAR1* siRNA. 60 hours after transfection, RNA extracts were generated and subjected to RNA-Seq. Color mapping indicates z-scores of gene expression based on RNA-Seq. *n*=3 biological replicates. **J.** Western blot of SUM149 *BRCA1*-Mut and *BRCA1*-Rev cells subjected to co-transfection with *ADAR1* siRNA and one of a series of siRNAs targeting pattern recognition receptors, as described in Fig. 6G, H. Cells were transfected with control, non-targeting siRNA (siCTRL), or with *ADAR1* siRNA SMARTpool and equimolar concentrations of *RIG1*, *MDA5*, *LGP2*, *PKR* or *cGAS* siRNA SMARTpools. 72 hours after transfection, cell lysates were generated and western blotted to detect RIG1, MDA5, LGP2, PKR and cGAS proteins. Data

representative of *n*=2 biologically-independent experiments. Short and long exposure times were used to image some membranes. **K-M.** Clonogenic survival of SUM149 *BRCA1*-Mut (K, L) and *BRCA1*-Rev (K, M) cells subjected to co-transfection with *ADAR1* siRNA and one of a series of siRNAs targeting pattern recognition receptors. Cells were transfected with control, non-targeting siRNA (siCTRL) or *ADAR1* siRNA SMARTpool, and with equimolar concentrations of *RIG1, MDA5, LGP2, PKR, cGAS* or *IFNAR1* siRNA SMARTpools. After transfection, cells were continuously cultured for 10 days, after which colonies were stained and counted. *PLK1*-targeting siRNA (siPLK1) was used as a positive control. Violin plots indicate median, lower and upper quartiles; N=6 values from individual wells, representative of *n*=2 biologically-independent experiments, two-way ANOVA post hoc Dunnett's test. **N.** Western blot of SUM149 *BRCA1*-Mut and *BRCA1*-Rev cells transduced with a doxycycline-inducible *ADAR1*-targeting shRNA in the context of exposure to the JAK/STAT pathway inhibitor (JSPi) ruxolitinib. Cells were either mock-transduced, or transduced with an *ADAR1*-targeting shRNA and subsequently exposed to doxycycline (50 ng/mL) in the presence or absence of mock control (DMSO) or ruxolitinib (10 µM). 72 hours after addition of doxycycline/ruxolitinib, cell lysates were generated and western blotted to detect RIG1, MDA5, LGP2, phosphorylated PKR (p-PKR), PKR, phosphorylated IRF3 (p-IRF3), and IRF3 proteins. Data representative of *n*=2 biologically-independent experiments. *P*-values, *<0.05, **<0.01, ***<0.001, ****<0.0001; ns, not significant. Source data are provided as a Source Data file.

SUPPLEMENTARY FIGURE 9

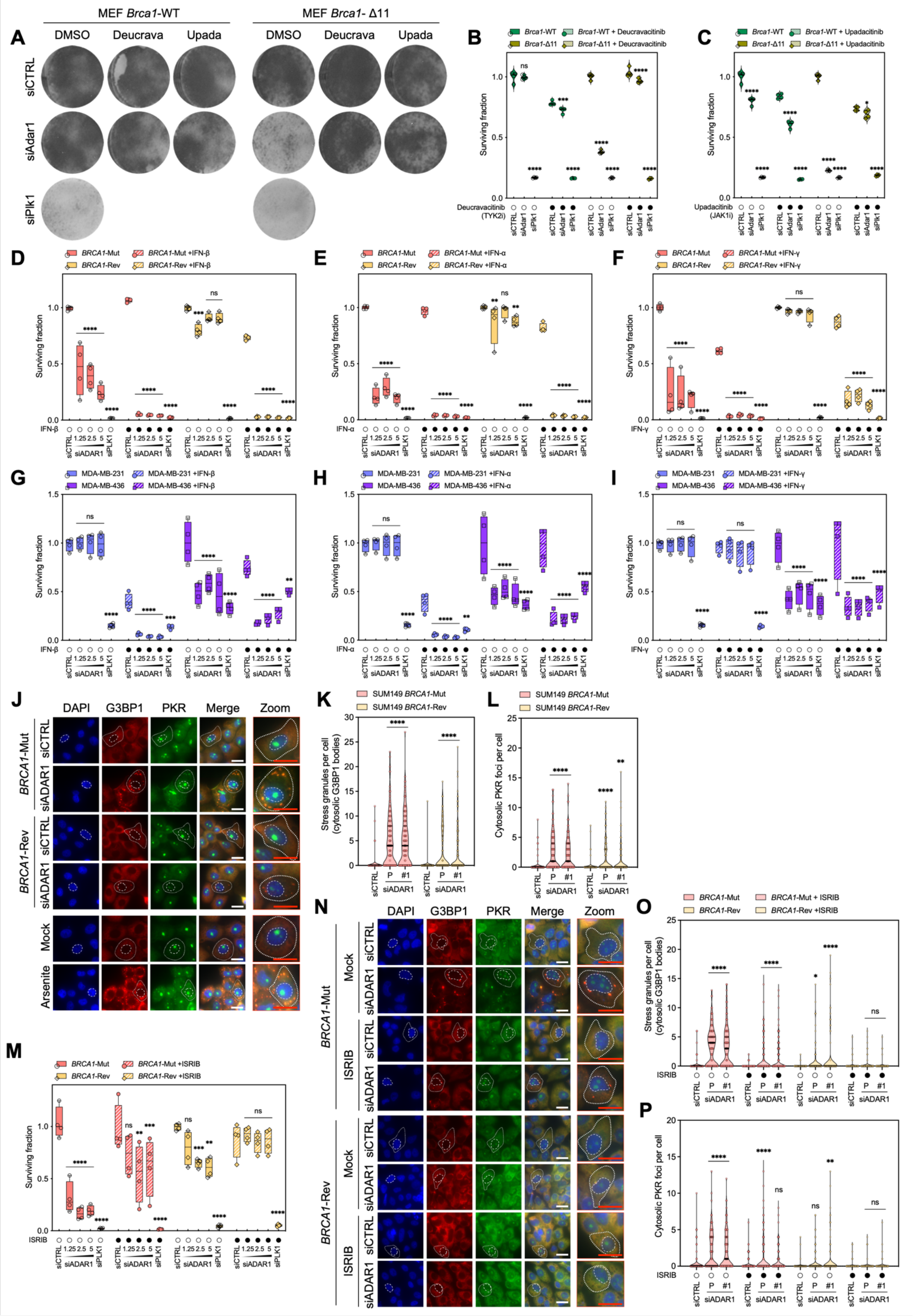

Supplementary Figure 9. Role of interferons and activation of the integrated stress response in the *BRCA1–ADAR1* synthetic lethal effects.

A-C. Clonogenic survival of MEF *Brca1*-wildtype (WT) and *Brca1*-mutant (Δ11) cells transfected with *Adar1* siRNA in the context of exposure to the JAK/STAT pathway inhibitors (JSPi) deucravacitinib (A, B) or upadacitinib (A, C). Cells were either transfected with control, non-targeting siRNA (siCTRL) or with *Adar1* siRNA SMARTpool. 24 hours after transfection, cells were exposed to mock control (DMSO), deucravacitinib (3 µM) or upadacitinib (5 µM) and continuously cultured for 7 days, after which they were stained and dissolved in acetic acid for quantification. *PLK1*-targeting siRNA (siPLK1) was used as a positive control. Violin plots indicate median, lower and upper quartiles; N=4 values from individual wells, representative of *n*=3 biologically-independent experiments, two-way ANOVA post hoc Dunnett's test. **D-F.** Cell survival of SUM149 *BRCA1*-Mut and *BRCA1*-Rev cells transfected with *ADAR1* siRNA in the context of exposure to interferon-β (D), interferon-α (E) or interferon-γ (F). 24 hours after transfection, cells were exposed to mock control (culture medium), interferon-β (IFN-β, 500 pg/mL), interferon-α (IFN-α, 25 ng/mL) or interferon-γ (IFN-γ, 500 pg/mL) and continuously cultured for 7 days, after which cell viability was determined by CellTiter-Glo®. Box-and-whiskers indicate median, lower and upper quartiles, and the min to max range; N=4 values from individual wells, representative of *n*=2 biologically-independent experiments, two-way ANOVA post hoc Dunnett's test. **G-I.** Cell survival of MDA-MB-231 and MDA-MB-436 cells transfected with *ADAR1* siRNA, in the context of exposure to interferon-β (G), interferon-α (H) or interferon-γ (I). Cells were either transfected with control, non-targeting siRNA (siCTRL) or with a titration of *ADAR1* siRNA SMARTpool (nM). 24 hours after transfection, cells were exposed to mock control (culture medium), interferon-β (IFN-β, 500 pg/mL), interferon-α (IFN-α, 25 ng/mL) or interferon-γ (IFN-γ, 500 pg/mL) and continuously cultured for 7 days, after which cell viability was determined by CellTiter-Glo®. *PLK1*-targeting siRNA (siPLK1) was used as a positive control. Box-and-whiskers indicate median, lower and upper quartiles, and the min to max range; N=4 values from individual wells, representative of *n*=2 biologically-independent experiments, two-way ANOVA post hoc Dunnett's test. **J-L.** Representative immunofluorescence images (J) and associated quantifications of G3BP1 bodies (K) and PKR foci (L) in SUM149 *BRCA1*-Mut and *BRCA1*-Rev cells transfected with *ADAR1* siRNA. Cells were transfected with control, non-targeting siRNA (siCTRL), or with either *ADAR1* siRNA SMARTpool (P) or an individual *ADAR1* siRNAs (#1). 72 hours after transfection, cells were fixed and stained with DAPI, G3BP1 and PKR immunostainings. Data are presented as the number of G3BP1 bodies or PKR foci per cell. Violin plots indicate median, lower and upper quartiles; N=150 values from individual cells, representative of *n*=2 biologically-independent experiments, two-way ANOVA post hoc Dunnett's test. **M.** Cell survival of SUM149 *BRCA1*-Mut and *BRCA1*-Rev cells transfected with *ADAR1* siRNA in the context of exposure to the integrated stress response pathway inhibitor ISRIB. Cells were either transfected with control, non-targeting siRNA (siCTRL) or with a titration of *ADAR1* siRNA SMARTpool (nM). 24 hours after transfection, cells were exposed to

mock control (DMSO) or ISRIB (10 μM) and continuously cultured for 7 days, after which cell viability was determined by CellTiter-Glo®. *PLK1*-targeting siRNA (siPLK1) was used as a positive control. Box-and-whiskers indicate median, lower and upper quartiles, and the min to max range; N=4 values from individual wells, representative of *n*=3 biologically-independent experiments, two-way ANOVA post hoc Dunnett's test. **N-P.** Representative immunofluorescence images (N) and associated quantifications of G3BP1 bodies (O) and PKR foci (P) in SUM149 *BRCA1*-Mut and *BRCA1*-Rev cells transfected with *ADAR1* siRNA in the context of exposure to the integrated stress response pathway inhibitor ISRIB. Cells were transfected with control, non-targeting siRNA (siCTRL), or with either *ADAR1* siRNA SMARTpool (P) or an individual *ADAR1* siRNAs (#1). 6 hours after transfection, cells were exposed to mock control (DMSO) or ISRIB (10 μM) and continuously cultured for 72 hours, after which cells were fixed and stained with DAPI, G3BP1 and PKR immunostainings. Data are presented as the number of G3BP1 bodies or PKR foci per cell. Violin plots indicate median, lower and upper quartiles; N=150 values from individual cells, representative of *n*=2 biologically-independent experiments, two-way ANOVA post hoc Dunnett's test. *P*-values, *<0.05, **<0.01, ***<0.001, ****<0.0001; ns, not significant. Source data are provided as a Source Data file.

SUPPLEMENTARY FIGURE 10

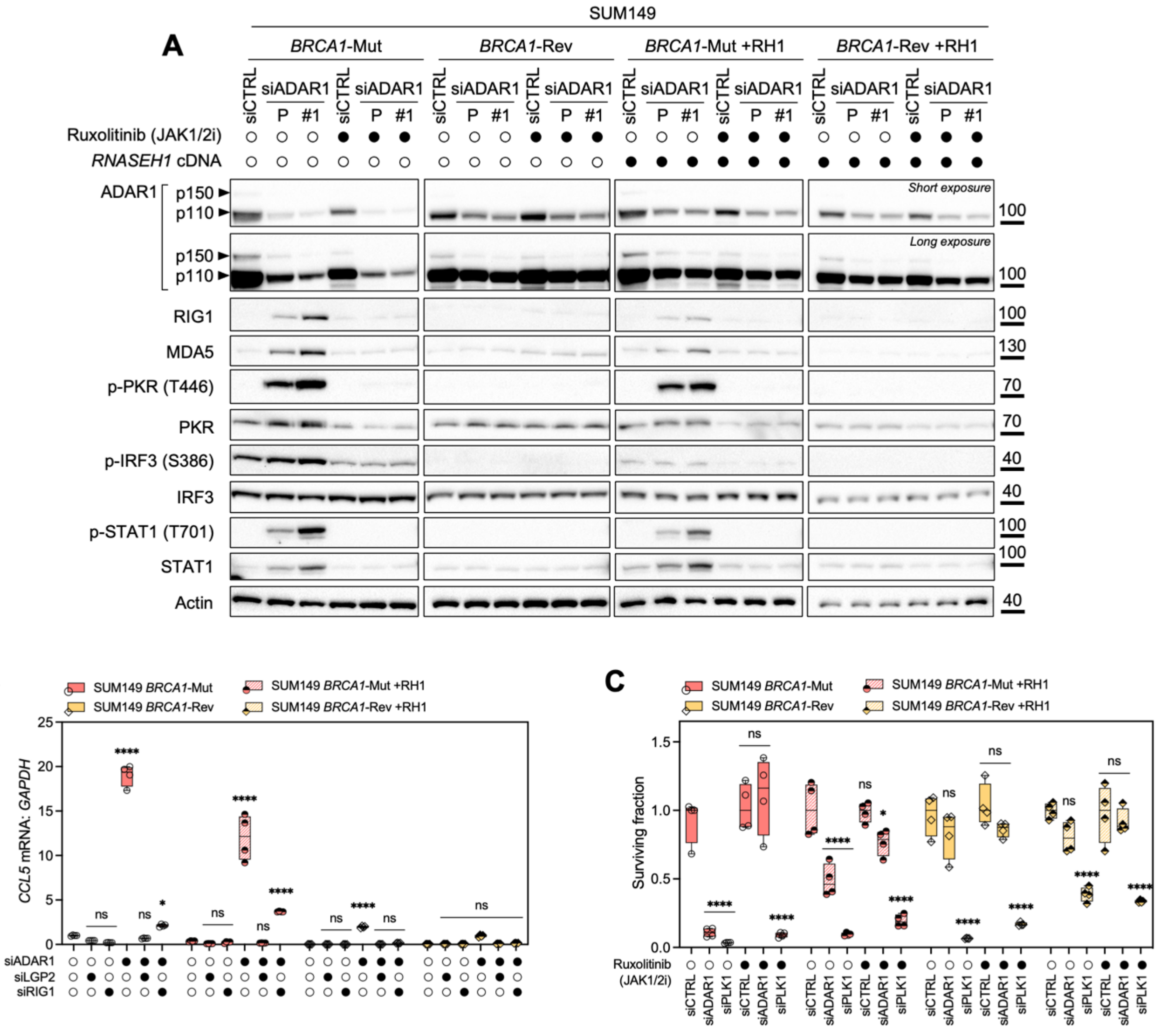

Supplementary Figure 10. Link between R-loops, pattern recognition receptors and the type I interferon response in the context of *BRCA1–ADAR1* synthetic lethality.

A. Western blot of SUM149 *BRCA1*-Mut and *BRCA1*-Rev cells transfected with *ADAR1* siRNA in the context of exogenous overexpression of RNase H1 (RH1) and exposure to the JAK/STAT pathway inhibitor (JSPi) ruxolitinib. Cells were transduced with a plasmid construct containing *RNASEH1* cDNA to induce stable overexpression of RNase H1, and were transfected with control, non-targeting siRNA (siCTRL), or with either *ADAR1* siRNA SMARTpool (P) or an individual *ADAR1* siRNAs (#1). 6 hours after transfection, cells were exposed to mock control (DMSO) or ruxolitinib (10 µM) and continuously cultured 72 hours, after which cell lysates were generated and western blotted to detect RIG1, MDA5, phosphorylated PKR (p-PKR), PKR, phosphorylated IRF3 (p-IRF3), IRF3, phosphorylated STAT1 (p-STAT1), and STAT1 proteins. Data representative of *n*=2 biologically-independent experiments. Short and long exposure times were used to image some membranes. **B.** RT-qPCR of *CCL5* mRNAs in SUM149 *BRCA1*-Mut and *BRCA1*-Rev cells subjected to co-transfection with *ADAR1* siRNA and *RIG1* or *LGP2* siRNA in the context of exogenous overexpression of RNase H1 (RH1). Cells were transduced with a plasmid construct containing *RNASEH1* cDNA to induce stable overexpression of RNase H1, and were transfected with control, non-targeting siRNA (siCTRL) or with *ADAR1* siRNA SMARTpool, and with equimolar concentrations of *RIG1* or *LGP2* siRNA SMARTpools. 72 hours after transfection, RNA extracts were generated and subjected to RT-qPCR. *CCL5* mRNAs were analyzed separately relative to *GAPDH*. Box-and-whiskers show arbitrary units of gene expression normalized to the *BRCA1*-Mut siCTRL condition, and indicate median, lower and upper quartiles, and the min to max range; N=4 values from individual measurements, representative of *n*=2 biologically-independent experiments, two-way ANOVA post hoc Dunnett's test. **C.** Cell survival of SUM149 *BRCA1*-Mut and *BRCA1*-Rev cells transfected with *ADAR1* siRNA in the context of exogenous overexpression of RNase H1 (RH1) and exposure to the JAK/STAT pathway inhibitor (JSPi) ruxolitinib. Cells were transduced with a plasmid construct containing *RNASEH1* cDNA to induce stable overexpression of RNase H1, and were transfected with control, non-targeting siRNA (siCTRL), or with *ADAR1* siRNA SMARTpool. 24 hours after transfection, cells were exposed to mock control (DMSO) or ruxolitinib (10 µM) and continuously cultured for 7 days, after which cell viability was determined by CellTiter-Glo®. *PLK1*-targeting siRNA (siPLK1) was used as a positive control. Box-and-whiskers indicate median, lower and upper quartiles, and the min to max range; N=4 values from individual wells, representative of *n*=3 biologically-independent experiments, two-way ANOVA post hoc Dunnett's test. *P*-values, *<0.05, **<0.01, ***<0.001, ****<0.0001; ns, not significant. Source data are provided as a Source Data file.
