# Supplementary material for: Autocrine interferon poisoning mediates ADAR1-dependent synthetic lethality in BRCA1/2-mutant cancers
Source: Nat Commun. 2025 Jul 29;16:6972. doi: 10.1038/s41467-025-62309-5 (PMC12307730; doi:10.1038/s41467-025-62309-5)
Supplement: Supplementary file 2 — Reporting Summary [file 41467_2025_62309_MOESM2_ESM.pdf]

nature portfolio

Corresponding author(s): Roman M. Chabanon, Sophie Postel-Vinay, Christopher J. Lord

Last updated by author(s): Jun 18, 2025

Reporting Summary

Nature Portfolio wishes to improve the reproducibility of the work that we publish. This form provides structure for consistency and transparency in reporting. For further information on Nature Portfolio policies, see our Editorial Policies and the Editorial Policy Checklist.

Statistics

For all statistical analyses, confirm that the following items are present in the figure legend, table legend, main text, or Methods section.

n/a	Confirmed	
☐	☒	The exact sample size (*n*) for each experimental group/condition, given as a discrete number and unit of measurement
☐	☒	A statement on whether measurements were taken from distinct samples or whether the same sample was measured repeatedly
☐	☒	The statistical test(s) used AND whether they are one- or two-sided<br>*Only common tests should be described solely by name; describe more complex techniques in the Methods section.*
☒	☐	A description of all covariates tested
☐	☒	A description of any assumptions or corrections, such as tests of normality and adjustment for multiple comparisons
☐	☒	A full description of the statistical parameters including central tendency (e.g. means) or other basic estimates (e.g. regression coefficient) AND variation (e.g. standard deviation) or associated estimates of uncertainty (e.g. confidence intervals)
☐	☒	For null hypothesis testing, the test statistic (e.g. *F*, *t*, *r*) with confidence intervals, effect sizes, degrees of freedom and *P* value noted<br>*Give P values as exact values whenever suitable.*
☒	☐	For Bayesian analysis, information on the choice of priors and Markov chain Monte Carlo settings
☒	☐	For hierarchical and complex designs, identification of the appropriate level for tests and full reporting of outcomes
☒	☐	Estimates of effect sizes (e.g. Cohen's *d*, Pearson's *r*), indicating how they were calculated

Our web collection on statistics for biologists contains articles on many of the points above.

Software and code

Policy information about availability of computer code

Data collection	Microsoft Excel (v16.89.1); Tecan SparkControl (v1.2); Biorad ImageLab (v6.1); Thermo Fisher QuantStudio (v1.6.1); Sartorius IncuCyte S3 (v6.2.9200.0); Olympus OlyVIA (v4.1).
Data analysis	GraphPad Prism (v10.3.1); Microsoft Excel (v16.89.1); ImageJ (v1.54); RStudio (v2024.09.0+375)

For manuscripts utilizing custom algorithms or software that are central to the research but not yet described in published literature, software must be made available to editors and reviewers. We strongly encourage code deposition in a community repository (e.g. GitHub). See the Nature Portfolio guidelines for submitting code & software for further information.

Data

Policy information about availability of data

All manuscripts must include a data availability statement. This statement should provide the following information, where applicable:

- Accession codes, unique identifiers, or web links for publicly available datasets
- A description of any restrictions on data availability
- For clinical datasets or third party data, please ensure that the statement adheres to our policy

Data availability statement (as written in the manuscript)
Materials availability
All new unique reagents generated in this study are available from the corresponding authors with a completed materials transfer agreement.

Dataset availability
The RNA-Seq data generated as part of this study have been deposited in the European Genome-Phenome Archive (EGA), under accession number EGAS50000000518 [https://ega-archive.org/studies/EGAS50000000518]. The datasets on EGA will be made available to interested researchers under limited access on a project-specific basis, subject to approval by the Gustave Roussy Data Access Committee in compliance with the data access agreement terms. Requests should be directed to the corresponding authors. Upon establishment of the data transfer agreement, EGA data release can be expected within 3 business days. Once access has been granted, the period for which the data can be downloaded is flexible and will be defined according to the downloader's needs.
The remaining data are available within the Article, Supplementary Information or Source Data file.
This study does not report original code.
Any additional information required to reanalyze the data reported in this study is available from the corresponding authors upon request.
Other resources availability
Requests for further information and resources should be directed to the corresponding authors: Roman M. Chabanon (roman.chabanon@gustaveroussy.fr), Sophie Postel-Vinay (sophie.postel-vinay@gustaveroussy.fr), Chris Lord (chris.lord@gustaveroussy.fr).

Research involving human participants, their data, or biological material

Policy information about studies with human participants or human data. See also policy information about sex, gender (identity/presentation), and sexual orientation and race, ethnicity and racism.

Reporting on sex and gender	Clinical specimens (tumor biopsies or surgical pieces) from patients with triple-negative breast cancer were used in this study for ADAR1 expression analyses by immunohistochemistry. Given that triple-negative breast cancer occurs in the overwhelming majority of cases in women, all the samples collected in this study are from female (sex) patients.
Reporting on race, ethnicity, or other socially relevant groupings	Data regarding race, ethnicity or any other socially-relevant groupings were not collected, and not considered in the study design.
Population characteristics	All female patients aged over 18 years with breast cancer and known gBRCA status, including luminal A, luminal B or triple-negative breast cancer, who underwent a tumour biopsy or surgical removal of their tumour. For patients treated with chemotherapy or PARP inhibitors in the neo-adjuvant or adjuvant setting, material from tumour biopsies at baseline (priori treatment initiation) and upon treatment may be used to assess dynamic changes over time. FNA samples are not be eligible.
Recruitment	Inclusion criteria:<br>1. Non-opposition to inclusion in the study.<br>2. Aged 18 or more.<br>3. Histologically confirmed invasive breast carcinoma, confirmed by the local pathologist.<br>4. Stage I-III at initial diagnosis and eligible to curative treatment and for whom initial tumour biopsy or surgical sample, collected before any treatment, is available.<br>5. Genetic testing confirming the presence or absence of gBRCA mutation.<br><br>Exclusion criteria:<br>1. Patients with personal history of other solid or haematological malignancies.<br>2. Patients with personal history of inflammatory disorders, restricted to genetically-defined autoimmune disorders.<br>3. Patients opposed to the use of their data for research.
Ethics oversight	Use of breast cancer samples was approved by the institutional ethics review board at Gustave Roussy (CSET, Commission Scientifique des Essais Thérapeutiques), in accordance with the Declaration of Helsinki. Informed consent was obtained from all subjects for the use of their tumor tissue as part of this study.

Note that full information on the approval of the study protocol must also be provided in the manuscript.

Field-specific reporting

Please select the one below that is the best fit for your research. If you are not sure, read the appropriate sections before making your selection.

[x] Life sciences [ ] Behavioural & social sciences [ ] Ecological, evolutionary & environmental sciences

For a reference copy of the document with all sections, see nature.com/documents/nr-reporting-summary-flat.pdf

Life sciences study design

All studies must disclose on these points even when the disclosure is negative.

Sample size	No statistical methods were used to pre-determine sample size. Sample sizes (at least three biologically-independent experimental replicates in most experiments, unless indicated otherwise) were chosen based on standard practices of the field.
Data exclusions	No data was excluded from this study.
Replication	The data presented in this study were successfully replicated in at least two biologically-independent experiments. Details of the replication are included in the figure legends for each individual experiment.

Randomization	Experiments were not randomized.
Blinding	Experiments were not blinded.<br>Blinding was not usually possible in cell culture experiments due to obvious differences in cell morphology and experimental outcome between groups.

Reporting for specific materials, systems and methods

We require information from authors about some types of materials, experimental systems and methods used in many studies. Here, indicate whether each material, system or method listed is relevant to your study. If you are not sure if a list item applies to your research, read the appropriate section before selecting a response.

Materials & experimental systems

n/a	Involved in the study	
☐	☒	Antibodies
☐	☒	Eukaryotic cell lines
☒	☐	Palaeontology and archaeology
☐	☒	Animals and other organisms
☒	☐	Clinical data
☒	☐	Dual use research of concern
☒	☐	Plants

Methods

n/a	Involved in the study	
☒	☐	ChIP-seq
☒	☐	Flow cytometry
☒	☐	MRI-based neuroimaging

Antibodies

Antibodies used	ADAR1 (p110, p150) - WB - Cell Signaling Technologies #14175 1:1,000<br>ADAR1 (p110, p150) - IHC - Santa Cruz #sc-73408 1:700<br>ADAR1 (p150) - IHC - Bethyl Laboratories #A303-883A 1 :1,000<br>β-Actin - WB - Sigma Aldrich #A1978 1:10,000<br>BRCA1 - WB - Cell Signaling Technologies #9010 1:1,000<br>BRCA2 - WB - Cell Signaling Technologies #10741 1:1,000<br>Casp3 - WB - Cell Signaling Technologies #9668 1:1,000<br>c-Casp3 (N175) - WB - Cell Signaling Technologies #9661 1:1,000<br>CCNA2 - IF - Abcam #ab181591 1:1,000<br>CGAS - WB - Cell Signaling Technologies #15102 1:1,000<br>CHK1 - WB - Cell Signaling Technologies #2360 1:1,000<br>p-CHK1 (S345) - WB - Cell Signaling Technologies #2341 1:1,000<br>eIF2α - WB - Cell Signaling Technologies #5324 1:1,000<br>p-eIF2α (S51) - WB - Cell Signaling Technologies #3398 1:1,000<br>Fibrillarin - IF - Abcam #ab166630 1:4,000<br>G3BP1 - WB, IF - Cell Signaling Technologies #61559 1:1,000<br>GAPDH - WB - Cell Signaling Technologies #2118 1:1,000<br>γ-H2AX (S139) - WB, IF - Merck #05-636 1:1,000<br>IRF3 - WB (Hs) - Abcam #ab76409 1:1,000<br>IRF3 - WB (Mm) - Cell Signaling Technologies #4302 1:1,000<br>p-IRF3 (S396) - WB (Hs) - Abcam #ab76493 1:1,000<br>p-IRF3 (S396) - WB (Mm) - Cell Signaling Technologies #29047 1:1,000<br>LGP2 - WB (Hs) - Cell Signaling Technologies #12869 1:1,000<br>LGP2 - WB (Mm) - Abcam #67270 1:1,000<br>MDA5 - WB - Cell Signaling Technologies #5321 1:1,000<br>c-PARP1 (N214) - WB - Cell Signaling Technologies #9141 1:1,000<br>PKR - WB - Cell Signaling Technologies #12297 1:1,000<br>PKR - IF - Santa Cruz #sc-6282 1:500<br>p-PKR (T446) - WB - Abcam #ab32036 1:1,000<br>RAD51 - IF - Abcam #ab133534 1:1,000<br>RIG1 - WB - Cell Signaling Technologies #3743 1:1,000<br>RNase H1 - WB - Santa Cruz #sc-376326 1:500<br>RNA:DNA hybrids - IF - Kerafast #ENH001 1:500<br>RPA - IF - Abcam #ab2175 1:1,000<br>STAT1 - WB (Hs) - Cell Signaling Technologies #9176 1:1,000<br>STAT1 - WB (Mm) - Cell Signaling Technologies #14994 1:1,000<br>p-STAT1 (T701) - WB - Cell Signaling Technologies #9167 1:1,000
Validation	Unless indicated otherwise, all antibodies used in this study were validated externally by the suppliers for each corresponding application. More information can be accessed on the suppliers websites: Cell Signaling Technologies (https://www.cellsignal.com/);

	Santa Cruz (https://www.scbt.com/home/); Abcam (https://www.abcam.com/); Bethyl Laboratories (https://www.fortislife.com/); Sigma Aldrich (https://www.sigmaaldrich.com/); Merck (https://www.merckmillipore.com/); Kerafast (https://www.kerafast.com/).<br><br>The ADAR1 (p110, p150; Santa Cruz #sc-73408) and ADAR1 (p150; Bethyl Laboratories #A303-883A) antibodies were validated in this study for use in immunohistochemistry, using knockout antibody validation in the HEK293T ADAR1-wildtype, ADAR1-knockout and ADAR1p150-knockout isogenic system.

Eukaryotic cell lines

Policy information about cell lines and Sex and Gender in Research

Cell line source(s)	SUM149 BRCA1-isogenic cells – described in Dréan et al. Mol. Cancer Ther. 2017 and Pettitt et al. Nat. Commun. 2018; original source: Asterand. RNase H1-overexpressing SUM149 cells were generated in this study.<br>MDA-MB-436 – source: ATCC. RNase H1-overexpressing MDA-MB-436 cells were generated in this study.<br>HCC1937 – source: ATCC<br>MDA-MB-231 – source: ATCC. RNase H1-overexpressing MDA-MB-231 cells were generated in this study.<br>CAL51 – source: DSMZ<br>CAL120 – source: ATCC<br>Hs578T – source: ATCC<br>RPE1 TP53–/– BRCA1-isogenic cells – described in Noordermeer et al. Nature 2018; gift from D. Durocher; original source: ATCC<br>DLD1 BRCA2-isogenic cells – source: Horizon Discovery<br>HEK293T ADAR1-isogenic – described in Chung et al. Cell 2018; gift from C. Rice; original source: ATCC<br>MEF Brca1-isogenic cells – described in Callen et al. Mol. Cell 2020; gift from A. Nussenzweig; original source: ATCC<br>4T1 Brca1/2-isogenic cells – described in Samstein et al. Nat. Cancer 2021; gift from R. Samstein; original source: ATCC<br>ID8 Brca1-isogenic cells – described in Walton et al. Sci. Rep. 2027; gift from I. McNeish; original source: Dr. K. Roby (University of Kansas Medical Center)
Authentication	All cell lines were authenticated by short-tandem-repeat (STR) typing using the GenePrint® 10 System (Promega, #B9510) to confirm identity prior to the study.<br>The BRCA1/2-gene status of isogenic and non-isogenic models was validated using functional assays (PARP inhibitor sensitivity, or BRCA1/2 expression).
Mycoplasma contamination	All cell lines used in this study were mycoplasma-negative. Mycoplasma testing was performed bimonthly using the MycoAlert Mycoplasma Detection Kit (Lonza, #LT07-318).
Commonly misidentified lines<br>(See ICLAC register)	No commonly misiendentified lines were used in this study.

Animals and other research organisms

Policy information about studies involving animals; ARRIVE guidelines recommended for reporting animal research, and Sex and Gender in Research

Laboratory animals	AB strain of zebrafish was used in this study.<br>Embryos were staged and cared for according to standard protocols, as described in Kimmel et al. Dev. Dyn. Off. Publ. Am. Assoc. Anat. 1995.
Wild animals	The study did not involve wild animals.
Reporting on sex	Sex was not considered in the study design.
Field-collected samples	The study did not involve samples collected from the field.
Ethics oversight	All zebrafish experiments were conducted in compliance with institutional animal protocols at Inserm, approved by DDPP Val de Marne, France under license number F 94-043-013.

Note that full information on the approval of the study protocol must also be provided in the manuscript.

Plants

Seed stocks	n/a
Novel plant genotypes	n/a
Authentication	n/a
